# New discoveries on the biology and detection of human chorionic gonadotropin

**DOI:** 10.1186/1477-7827-7-8

**Published:** 2009-01-26

**Authors:** Laurence A Cole

**Affiliations:** 1 USA hCG Reference Service, Obstetrics and Gynecology, and Biochemistry and Molecular Biology, University of New Mexico, Albuquerque NM, USA

## Abstract

Human chorionic gonadotropin (hCG) is a glycoprotein hormone comprising 2 subunits, alpha and beta joined non covalently. While similar in structure to luteinizing hormone (LH), hCG exists in multiple hormonal and non-endocrine agents, rather than as a single molecule like LH and the other glycoprotein hormones. These are regular hCG, hyperglycosylated hCG and the free beta-subunit of hyperglycosylated hCG.

For 88 years regular hCG has been known as a promoter of corpus luteal progesterone production, even though this function only explains 3 weeks of a full gestations production of regular hCG. Research in recent years has explained the full gestational production by demonstration of critical functions in trophoblast differentiation and in fetal nutrition through myometrial spiral artery angiogenesis.

While regular hCG is made by fused villous syncytiotrophoblast cells, extravillous invasive cytotrophoblast cells make the variant hyperglycosylated hCG. This variant is an autocrine factor, acting on extravillous invasive cytotrophoblast cells to initiate and control invasion as occurs at implantation of pregnancy and the establishment of hemochorial placentation, and malignancy as occurs in invasive hydatidiform mole and choriocarcinoma. Hyperglycosylated hCG inhibits apoptosis in extravillous invasive cytotrophoblast cells promoting cell invasion, growth and malignancy. Other non-trophoblastic malignancies retro-differentiate and produce a hyperglycosylated free beta-subunit of hCG (hCG free beta). This has been shown to be an autocrine factor antagonizing apoptosis furthering cancer cell growth and malignancy.

New applications have been demonstrated for total hCG measurements and detection of the 3 hCG variants in pregnancy detection, monitoring pregnancy outcome, determining risk for Down syndrome fetus, predicting preeclampsia, detecting pituitary hCG, detecting and managing gestational trophoblastic diseases, diagnosing quiescent gestational trophoblastic disease, diagnosing placental site trophoblastic tumor, managing testicular germ cell malignancies, and monitoring other human malignancies. There are very few molecules with such wide and varying functions as regular hCG and its variants, and very few tests with such a wide spectrum of clinical applications as total hCG.

## Background

In 1920 Hirose showed a hormonal link between a human placental hormone and progesterone production by corpus luteal cells [1]. The name human chorionic gonadotropin (hCG) was formulated for the hormone. The promotion of progesterone production by corpus luteal cells was assumed to be the principal function of this hormone. Until recent years this has been assumed to be the sole function for hCG.

The first pregnancy test, the rabbit test, was formulated [2,3] in the 1920s. For four decades bioassays such as the rabbit test were the only practical way to measure hCG or detect pregnancy. In 1960 with the development of polyclonal antibodies came the agglutination inhibition test [4]. Then, in 1967 with discovery of the competitive immunoassays the hCG radioimmunoassay was developed [5-8]. This became the first rapid and sensitive test and led to the dawn of commercial hCG tests as seen today. hCG testing became part of the evaluation of every pregnancy. The initial radioimmunuassays used an antibody to whole hCG α β dimer. The α-subunit of hCG is identical with the α-subunit of LH. As such the initial RIA detected both hCG and LH limiting its use for the early detection or pregnancy. In 1973 the hCG β-subunit radioimmunoassay was introduced, specifically detecting hCG through its β-subunit [9]. This led to sensitive and specific pregnancy tests, detecting pregnancy soon after missing menses. The discovery of monoclonal antibodies in 1975 was paramount to the development of modern immunometric tests [10]. Two-antibody immunometric assays for hCG arose in the nineteen eighties, and with them came sensitive antibody enzyme labeling and high sensitivity fluorimetric and chemiluminescent tracers [11-14]. These are the formats of assays used in commercial laboratories today.

**Figure 1:**
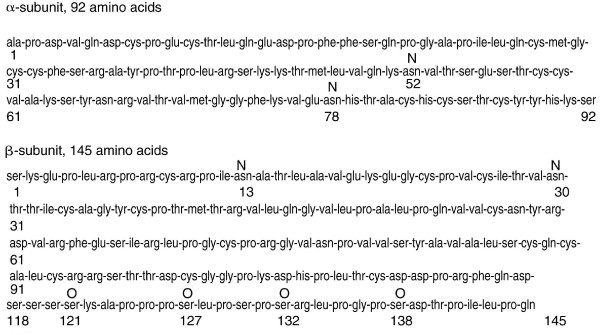
**Amino acid sequence of hCG α-subunit and β-subunit **[16,17]. Digits indicated amino acid residue positions and N and O indicate the positions of N- and O-linked oligosaccharides.

**Figure 2:**
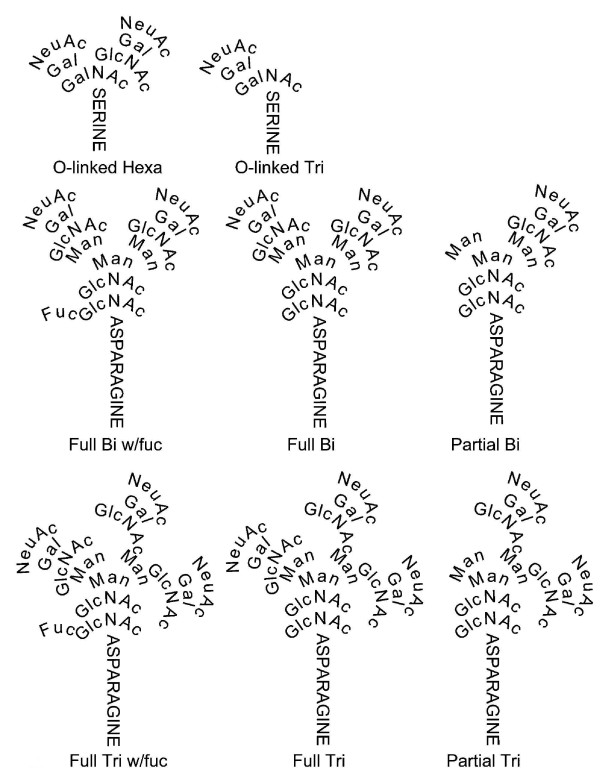
**Structures of O-linked hexa- and tris-saccharide and N-linked Bi (biantennary) and Tri (triantennary) oligosaccharides attached to regular hCG, hyperglycosylated hCG and hyperglycosylated hCG free β **[18-20]. NeuAc is N-acetylneuraminic acid or sialic acid, GalNAc in N-acetylgalactosamine, Gal is galactose, GlcNAc is N-acetylglucosamine, Man is mannose and Fuc is fucose.

In 1970 hCG was shown to be a non-covalently linked dimer [15]. The 1970s saw the determination of amino acid sequence of hCG subunits (Figure 1), and the observation that hCG contained 4 N-linked and 4 O-linked oligosaccharides [16,17]. The 1980s and 1990s saw the determination of the structures of the N- and O-linked oligosaccharides on hCG as produced in pregnancy and gestational trophoblastic diseases (Figure 2) [18,19], it saw the elaboration of the hCG subunit gene structures [20], and to our understanding of the hCG/LH receptor and the mechanisms of hCG endocrinology whereby hCG promotes progesterone production [21,22].

Was this the completion of the hCG physiology, biochemistry and immunoassay story? In the past 10 years a revolution has occurred with this molecule. Firstly, with the finding that the polypeptides that make hCG do not just make one biologically active molecule, but a group of three biologically important molecules, regular hCG, hyperglycosylated hCG and hyperglycosylated hCG free β-subunit. Each of these three molecules having different physiological functions and each having had separate roles in the evolution of humans. We have also seen in the last 10 years the elaboration of our understanding of pituitary hCG and new data on hCG and trophoblast function. Furthermore, there has been the discovery of numerous new applications when measuring only hyperglycosylated hCG and only the hyperglycosylated and regular free β-subunit. This review examines these hCG variant molecules and how the past 10 years they have changed the way they are viewed. How hCG is a key element in the evolution of the human brain, and how today we look differently at defined hCG variant assays and the specificity of total hCG assays in general.

## Occurrence

Regular hCG predominates in all normal and abnormal pregnancies (Table 1). It is the principal hCG variant produced through the bulk period of pregnancy (Table 2), it is present at reduced levels during spontaneous aborting and ectopic pregnancy [14] and at double regular levels in Down syndrome pregnancy [23,24]. Regular hCG is also the principal molecule produced in individuals with hydatidiform moles or pregnancies comprising solely trophoblast tissue [18,25]. Regular hCG is also normally produced by the pituitary gland at the time of LH peaks and in menopausal women [25,26].

**Table 1:** Occurrence of regular hCG, hyperglycosylated hCG and hyperglycosylated hCG free β in serum and urine samples [30-34,41-43,45-59].

	Regular hCG	Hyperglycosylated hCG	Hyperglycosylated hCG free β
1. Pregnancy			
Early pregnancy (3–5 weeks)	±	✓✓✓	✓
General Pregnancy (6 weeks – term)	✓✓✓	✓	✓
Biochemical pregnancy	✓✓✓	± MK	±
Spontaneous abortion	✓✓✓	± MK	±
Ectopic pregnancy	✓✓✓	± MK	±
Down syndrome pregnancy	✓✓✓ MK	✓ MK	✓ MK
Preeclampsia	✓✓✓	± MK	±
Pituitary	✓✓✓	X	✓
			
2. Neoplasia			
Hydatidiform mole	✓✓✓	✓	±
Invasive mole	✓	✓	✓
Choriocarcinoma	±	✓✓✓ MK	✓
Quiescent trophoblastic disease	✓✓✓	X MK	X
Placental site trophoblastic tumor	✓	±	✓✓✓ MK
Testicular germ cell tumor	±	✓✓✓ MK	✓
Non-gestational malignancies	±	±	✓✓✓ MK

All variants are present in both serum and urine samples. In this table, ✓✓✓ indicates principal hCG-form detected, ✓ indicates a key hCG-form always present, ± indicates absent or detected at very low levels, X indicates complete absence. ✓✓✓ MK and ✓ MK indicate elevated concentration is a marker for disorder, ± MK and X MK indicate deficiency or absence is a marker for disorder.

**Table 2:** Detection of total hCG, hyperglycosylated hCG and hyperglycosylated hCG free β in pregnancy serum [43,45-48].

Gestation age (weeks since last menses)	N	Total hCG Median (mIU/ml)	Hyperglycosylated hCG (%) mean ± SD	Hyperglycosylated hCG free β (%) mean ± SD
3 weeks (implantation)	n = 5	22	89 ± 24%	12 ± 22%
4 weeks (missing menses)	n = 16	239	49 ± 21%	7.3 ± 5.9%
5 weeks	n = 27	3,683	36 ± 13%	1.7 ± 0.75%
6 weeks	n = 25	16,850	21 ± 14%	1.4 ± 0.63%
7 weeks	n = 22	32,095	16 ± 13%	1.0 ± 0.24%
8 weeks	n = 33	95,282	7.0 ± 5.4%	0.99 ± 0.51%
9 weeks	n = 7	128,300	5.1 ± 4.4%	0.92 ± 0.35%
10 weeks	n = 8	102,750	4.3 ± 3.1%	0.68 ± 0.47%
11 – 13 weeks	n = 21	95,650	2.3 ± 1.5%	0.67 ± 0.33%
14 – 17 weeks	n = 57	32,275	1.3 ± 0.61%	0.62 ± 0.26%
18 – 26 weeks	n = 62	21,250	0.65 ± 0.60%	0.55 ± 0.42%
27 – 40 weeks	n = 49	21,025	0.36 ± 0.16%	0.47 ± 0.19%

Proportion hyperglycosylated hCG and hyperglycosylated hCG free β is the concentrations measured as a proportion of total hCG. SD is standard deviation. Gestational age is 3 day plus and 3 days minus specified week. So that data for 6 weeks pregnancy is 5 weeks 4 days to 6 weeks 3 days.

**Figure 3:**
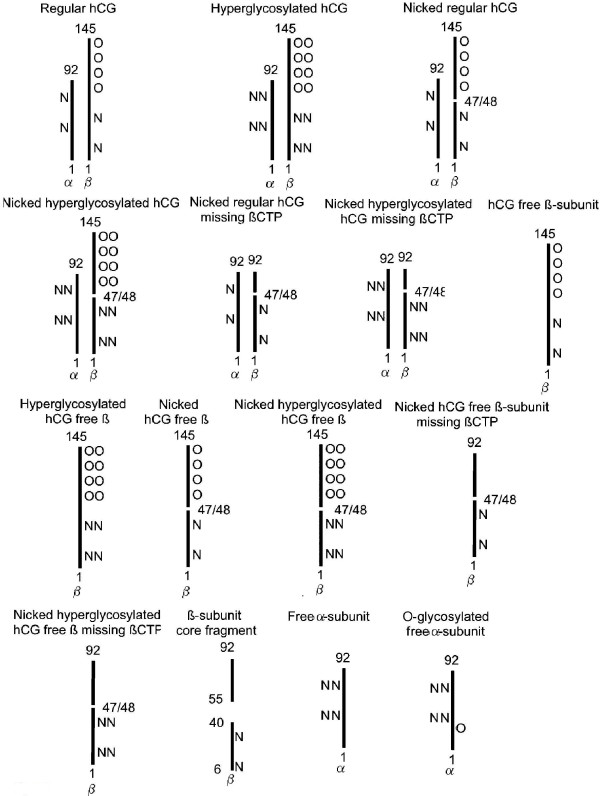
**Outline of the structures of the 15 common hCG variants present in serum and urine samples in either pregnancy, gestational trophoblastic disease or other malignancy**. Numbers refer to subunit polypeptide amino acid numbers (as in 1 and 145 in the 145 amino acid long β-subunit), O refers to O-linked and N to N-linked oligosaccharides. OO and NN refer to large or hyperglycosylated oligosaccharides. α is α-subunit and β is β-subunit. βCTP is the C-terminal segment (residues 93–145) on the regular or hyperglycosylated hCG β-subunit.

Multiple variants of hCG have been detected in serum and urine samples (Figure 3). A free α-subunit of hCG and a free β-subunit of hCG (hCG free β) have been demonstrated in pregnancy serum, cancer patient serum and urine and cancer cell line culture fluids (Table 1) [27,28]. A terminal degradation variant of hCG β-subunit is found in urine samples. This is β-subunit core fragment, it comprises two fragment of β-subunit, β6–40 and β55–92, held together by disulfide bonds (Figure 3) [29]. Urine β-core fragment is used as a general tumor marker for all non-gestational malignancies [30-34]. A large form of hCG dimer is synthesized by choriocarcinoma cells [15,18,35]. This is hyperglycosylated hCG with 1.5 fold larger N-linked and double size O-linked oligosaccharides (Figure 3) [18,19,36]. Similar unduly large N- and O-linked oligosaccharides have been demonstrated on the hCG free β produced by non-gestational cancer cells, we call this molecule hyperglycosylated hCG free β [20,35], Similar large oligosaccharides are found on the α-subunit secreted in pregnancy, free α-subunit [37]. Just as hCG is hyperglycosylated in choriocarcinoma and gets larger oligosaccharide side chains, free α-subunit is further glycosylated in the malignancy, gaining an additional O-linked oligosaccharide, we call this molecule O-glycosylated free α-subunit [38].

The regular hCG and hyperglycosylated hCG degradation pathways involve elastase and other proteases secreted by macrophages associated with tumor tissue or present in the circulation [39]. These cleave or nick hCG at β-subunit 44–45 or 47–48 [18,39], generating a nicked regular hCG and a nicked hyperglycosylated hCG, a nicked hCG free β and nicked hyperglycosylated hCG free β (Figure 3). These same enzymes progress further to cleave and release the C-terminal peptide on the nicked molecules (βCTP, β residues 93–145) generating nicked hCG missing βCTP, nicked hyperglycosylated hCG missing βCTP, nicked hCG free β missing βCTP and nicked hyperglycosylated free β missing βCTP [18,25,39]. This is 15 variants, all found in serum and urine samples in normal or abnormal pregnancies, gestational trophoblastic diseases or non-gestational malignancies (Figure 3).

**Figure 4:**
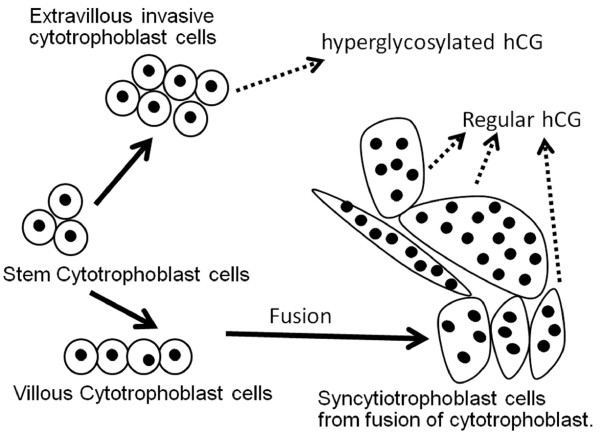
**The differentiation of trophoblast cells in placental villi **[54]. Fusion of villous cytotrophoblast cells is controlled by regular hCG [55]. Extravillous invasive cytotrophoblast cells produce hyperglycosylated hCG [44], while fused syncytiotrophoblast cells make regular hCG [40-42].

Of these 15 variants, just 5 are natural synthetic products made by the placenta in pregnancy or by non-trophoblastic malignancies, regular hCG, hyperglycosylated hCG, hyperglycosylated hCG free β and free α-subunit and O-glycosylated free α-subunit. The other 10 variants are degradational product, from macrophage cleavage and cleavage by proteases in the circulation and the kidney. Trophoblast cells are the active cells of the placenta (Figure 4). Fused trophoblast cells or syncytiotrophoblast produce the hormone regular hCG [40]. These same cells make an excess of α-subunit which is secreted as free α-subunit [37,40]. Initial studies indicated that cytotrophoblast cells hyperglycosylated hCG [40,41]. Hyperglycosylated hCG is an autocrine factor, it functions separate to regular hCG to promote invasion at implantation of pregnancy and malignancy in gestational trophoblastic diseases [41-44]. Recent studied by Handschuh and colleagues show that hyperglycosylated hCG is made only by the extravillous invasive cytotrophoblast cells [44], the cell that terminate anchoring villi and invade the myometrium. These same cells make an excess of α-subunit which is secreted as free α-subunit and O-glycosylated free α-subunit [37,40]. It is noteworthy that free α-subunit has no known biological function, it is a biological waste product [37].

Non-gestational malignancies produce hyperglycosylated free β [14,31-34], as demonstrated this acts as an autocrine factor directly promoting cancer cell growth and malignancy. Clearly, 3 of the 5 synthesized variants of hCG are independent molecules, hormones or autocrines, with separate functions, regular hCG, hyperglycosylated hCG and hyperglycosylated hCG free β.

Hyperglycosylated hCG is the extravillous invasion cytotrophoblast cell invasion signal [40-44], and is produced in states characterized by cytotrophoblast cells or invasion (Table 1). It is the principal or sole form of hCG produced in the first week of pregnancy, following implantation of the fetus (Table 2) [43,45-47]. Unduly low levels of hyperglycosylated hCG clearly mark failing pregnancies, whether a biochemical pregnancy or early pregnancy loss, spontaneous abortion or ectopic pregnancy [43,45-47]. While minimally present in hydatidiform mole cases, rising proportions of hyperglycosylated hCG mark invasive hydatidiform mole (invasive mole) and development and advancement of choriocarcinoma (Table 1) [41-44,48-50]. Hyperglycosylated hCG can also differentiate invasive and non-invasive (quiescent trophoblastic disease) gestational trophoblastic diseases [43,49]. Hyperglycosylated hCG similarly marks retrodifferentiation to cytotrophoblast cells and cancer advancement in testicular germ cell malignancy cases [42,48]. Hyperglycosylated hCG has also been used as an improved marker for fetuses with Down syndrome, in both the first and second trimesters of pregnancy [51,52]. It is also an outstanding marker for predicting third trimester hypertensive disorders and preeclampsia in the second trimester of pregnancy [53].

It is important to fully understand the differentiation of trophoblast cells which separate production of hyperglycosylated hCG and regular hCG (Figure 4). Stem cytotrophoblast cells differentiate to make extravillous invasive cytotrophoblasts [54], which produce hyperglycosylated hCG (Figure 4). They also differentiate to make villous cytotrophoblast cells which do not produce hyperglycosylated hCG(Figure 4). These fuse to form multinucleated syncytiotrophoblast cells, with 2 to 50 nulei [54]. The fusion of cytotrophoblast cells is controlled and promoted by regular hCG [55]. Cytotrophoblast cells are the principal cells present at the time of pregnancy implantation. These cells rapidly differentiate to extravillous invasive cytotrophoblast, and villous cytotrophoblast, which fuse to syncytiotrophoblast as pregnancy advances (Figure 4) [54,55]. In choriocarcinoma and testicular germ cell malignancies cytotrophoblast cell predominate. It appears that the carcinogenesis process le makes a non-differentiating non-villous invasive cytotrophoblast cells producing hyperglycosylated hCG [42]. Thus hyperglycosylated hCG is a potent marker for these malignancies. In Down syndrome pregnancies, trisomy 21 limits the differentiation of villopus cytotrophoblast cells leading to an accumulation of these cells and hyperglycosylated hCG [56]. This is apparently due to a failure of regular hCG-promoted differentiation [56]. It is for this reason that hyperglycosylated hCG is a marker of Down syndrome in the first and second trimester of pregnancy.

Hyperglycosylated hCG free β is produced at the time of implantation in very early pregnancy, and at very low proportions throughout the length of pregnancy (Table 2). While present at low levels, it is widely used in predicting Down syndrome in the first and second trimesters of pregnancy [57]. The hyperglycosylated hCG free β is produced in other or non-trophoblastic malignancies. A portion of almost every human malignancy advanced, retrodifferentiates and produces hyperglycosylated hCG free β [30-38,58-62]. Hyperglycosylated hCG free β production also distinguishes placental site trophoblastic tumors from other forms of gestational trophoblastic disease (Table 1).

All told, regular hCG, hyperglycosylated hCG and hyperglycosylated hCG free β are widespread in pregnancy, pregnancy abnormalities, trophoblastic malignancies and other or non-trophoblastic malignancies, with each form predominating in different disorders (Table 1). These 3 molecules plus ten degradation products constitute the 13 forms of hCG β-subunit present in serum or urine samples under different conditions (Figure 3). Ideally a total hCG tests should detect all of these hCG-related molecules to optimal monitor pregnancy, gestational trophoblastic diseases and cancer cases.

## Structure

The peptide structure of the hCG group of molecules was established by Bahl and colleagues in 1972 [63] and confirmed and refined by Morgan and colleagues in 1975 (Figure 1) [64]. Oligosaccharides constitute approximately 25–30% of the molecular weight of regular hCG. The N- and O-linked oligosaccharide structures of regular hCG were first determined by Kessler and colleagues [65,66], and refined and confirmed by Mizouchi and Kobata in 1980 [67], further refined by Elliott and colleagues in 1997 [18] and by Kobata and Takeuchi in 1999 [19]. As illustrated in Figure 1, regular hCG, molecular weight 36,000, comprises a 145 amino acid β-subunit and a 92 amino acid α-subunit. There are 2 N-linked oligosaccharides on the α-subunit of hCG and 2 N-linked oligosaccharides on the β-subunit of hCG. There are also 4 O-linked oligosaccharides on the C-terminal peptide region of the β-subunit of hCG (Figure 1).

Trisaccharide O-linked oligosaccharides are present on the C-terminal peptide segment of regular hCG (Figure 2) [18,19]. A Full Bi (biantennary) and a Partial Bi structure are present at the 2 N-linked oligosaccharide sites on the α-subunit of regular hCG and a Full Bi and Full Bi w/fuc biantennary structure are present at the 2 N-linked oligosaccharide sites on the β-subunit of regular hCG [18,19].

Between 1985 and 1997 Cole and colleagues examined the structure of the hCG produced in invasive trophoblastic disease, choriocarcinoma, and showed it to be different to that on regular hCG [18,68,69]. As found, this invasive cell hCG, whether from early pregnancy or choriocarcinoma had double size hexasaccharide O-linked oligosaccharides and larger (triantennary) N-linked oligosaccharides (Figure 2). This molecule was named hyperglycosylated hCG. No difference was observed in hCG peptide sequence, only in the oligosaccharide side chains [18]. Hexasaccharides replaced trisaccharides on O-linked oligosaccharides of hyperglycosylated hCG. Full Tri (triantennary), Full Tri w/fuc and Partial Tri structures replaced the Full Bi, Full Bi w/fuc and Partial Bi biantennary oligosaccharides at the N-linkage sites on hyperglycosylated hCG. These increased the molecular weight of hyperglycosylated hCG from approximately 36,000 (regular hCG) to approximate 40,500. The molecular weights are approximate (± 1000) due to variability in sialic acid content [18,19]. These oligosaccharide structures on regular and hyperglycosylated hCG have all now been confirmed by two other groups using alternate oligosaccharide structure methods [19,36,42,70].

Variations are found in the N-linked and O-linked oligosaccharide structures on hCG with differences in cellular metabolism and different expression of glycosyltransferase activities (18,19,36). In 1997 it was demonstrated that the 4 O-linked oligosaccharides are the principal difference between choriocarcinoma or testicular germ cell malignancy hyperglycosylated hCG and pregnancy regular hCG [18,42]. There are 2 principal types of O-linked oligosaccharides (Figure 2), trisaccharide and hexasaccharide. While first trimester normal pregnancy urine hCG contained 12.3 to 19% of the hexasaccharide sugar structure (regular hCG, n = 6 individuals, mean = 15.6%), choriocarcinoma urine hCG contains 60 to 100% hexasaccharide structures (hyperglycosylated hCG, n = 6 individuals, mean = 74.2%), 5-fold more than first trimester pregnancy [18]. A smaller difference was observed with the 4 N-linked oligosaccharide structures on pregnancy and choriocarcinoma molecules [18]. While 6 first trimester pregnancy samples contained an average of 10.3% triantennary structures at the four N-linked sites on hCG, 5 choriocarcinoma cases contained 33% of the triantennary structures (3-fold more than first trimester pregnancy) [18,19,42]. The largest difference between regular hCG and hyperglycosylated hCG is at the O-linked oligosaccharides.

In 2006 the oligosaccharides were evaluated for the first time on a site by site basis using mass spectrometry methods with multiple regular hCG and hyperglycosylated hCG preparations from pregnancy, choriocarcinoma and testicular germ cell malignancies [36]. Researchers found a greater fucose content on N-linked oligosaccharides in cancer cases [36]. They demonstrated site-specific difference in O-linked oligosaccharides. They showed the constant presence of predominantly hexasaccharide structures in pregnancy and choriocarcinoma at Serine residue 121 [68–89%], with variable structures on Ser 127, 132 and 138, primarily trisaccharide structures on regular hCG in pregnancy and hexasaccharide structures on hyperglycosylated hCG in choriocarcinoma [36]. In conclusion, combining results, it appears that differences in hCG β-subunit O-glycosylation at 3 sites, Ser 127, 132 and 138, are the principal discriminator of regular hCG and hyperglycosylated hCG [18,19,36].

Here we refer to pregnancy hCG as regular hCG and choriocarcinoma hCG as hyperglycosylated hCG. With the advent of hyperglycosylated hCG antibodies and very specific immunoassays [71,72], this larger form of hCG was identified in urine samples considered too dilute in hCG for analysis of oligosaccharide structures. By these methods, hyperglycosylated molecules were shown to be 96% of total hCG in urine samples and 89% in serum samples (Table 2) at the time of embryo implantation [43,45,46,73]. The proportions of hyperglycosylated hCG molecules rapidly decline in the weeks following implantation, averaging 68% and 49% in urine and serum samples at the time of missing menses, 50% and 36% at 5 weeks of gestation, 25% and 21% at 6 weeks of gestation, and < 1.5% in the second and third trimesters of pregnancy (Table 2) [45,46,74].

The free β-subunit of hCG detected in choriocarcinoma patient urine and in urine from individuals with non-trophoblastic neoplasm is larger than the β-subunit isolated from regular hCG dimer. Larger oligosaccharide side chains have been indicated [75-77]. Examination of the structure of the free β-subunit of hCG reveals molecules with a high proportion of fucosylated trianntenary N-linked oligosaccharide structures and hexasaccharide O-linked oligosaccharides, analogous to hyperglycosylated hCG [36]. This explains the larger molecular size for hCG free β observed in cancer cases. The name hyperglycosylated hCG free β is adopted for this molecule. It appears that the free α-subunit of hCG receives larger oligosaccharide side chains than the α-subunit of hCG dimer [37], and that the free β-subunit like the free α also is hyperglycosylated.

## Dissociation, cleavage and clearance

**Figure 5:**
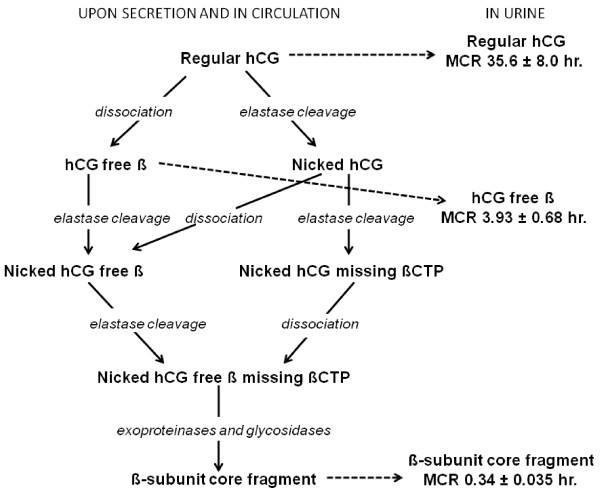
**The dissociation, degradation and clearance pathways of regular hCG **[39,78-87]. Metabolic clearance half-times (MCR) are those published by Wehman and colleagues [85-88]. Similar degradation and clearance pathways are predicted for hyperglycosylated hCG and hyperglycosylated hCG free β with an end product of urine β-subunit core fragment.

In the previous section we discussed the structures of the regular hCG, hyperglycosylated hCG, hyperglycosylated hCG free β and free α-subunit made by trophoblast and cancer cells. Here we discuss the dissociation, cleavage and clearance of these hCG forms, or the synthesis and structure of the hCG degradation intermediates. Figure 5 summarizes the dissociation, cleavage and circulatory clearance pathways of hCG, hyperglycosylated hCG and hyperglycosylated hCG free β [39,78-88].

As an initial step, the dimers regular hCG and hyperglycosylated hCG are either slowly cleaved (nicked) or slowly dissociated into α and β subunits (Figure 5). The dissociation half-time of dimers into subunits is 700 ± 78 hours at 37°C [78]. Hyperglycosylated hCG dissociates much more rapidly that regular hCG (140 ± hours at 37°C [39]).

A macrophage or circulating leukocyte elastase protease cleaves hCG β-subunit at Val residue 44 (residue 44–45 cleavage) or Gly residue 47 (residue 47–48 cleavage) generating nicked hCG (Figure 1) [39,78]. Nicked hCG is 30-fold less stable than regular hCG (dissociation half time 22 ± 5.2 hr. at 37°C [78]) rapidly dissociating releasing a free α-subunit and a nicked hCG free β. Similarly, dissociated hCG β-subunit is rapidly cleaved by leukocyte elastase to make nicked hCG free β. Both nicking and dissociation eliminate hCG hormone activity (shown by ability to bind corpus luteum hCG/LH receptor and to promote progesterone production [39]). Dimer is cleared from the circulation very slowly (clearance half-life 35.6 ± 8.0 hr. [82,84]), hCG free β is cleared 10-fold faster (3.93 ± 0.68 hr. [83,84]). As such the combination of nicking and dissociation, in either order, leads to the rapid deactivation and clearance of regular or hyperglycosylated hCG [78].

Nicked hCG or nicked hCG free β, while cleaved at 44–45 or 47–48, remain structurally intact due to 5 disulfide linkages between the component peptides. Further degradation of hCG by leukocyte elastase leads to cleavage of β-subunit at Leu residue 92 [39]. The peptide 93–145 the βCTP is not held by disulfide linkages and leads to nicked dimer missing the βCTP or nicked hCG free β missing βCTP [18,32,39]. Nicked dimer missing the βCTP is rapidly degraded to nicked hCG free β missing the βCTP (Figure 5). The nicked hCG free β missing the βCTP made by either pathway is rapidly degraded in the kidney by exoproteinases and glycosidases to β-subunit core fragment, the terminal degradation product of regular hCG, hCG free β, hyperglycosylated hCG and hyperglycosylated hCG free β [85-88]. β-subunit core fragment is β-subunit residues 6–40 linked to residues 55–92, held together by 5 disulfide linkages. It has degraded N-linked oligosaccharides, missing the non-reducing terminal sialic acid, galactose and N-acetylglucosamine residues and fucose residues so terminates in mannose [85]. As shown, injection of pure CHO cell recombinant regular hCG (contains no nicked, hyperglycosylated or dissociated hCG components) into the human circulation leads to excretion of β-subunit core fragment in the urine [88], clearly confirming the degradation pathway.

Nicking and cleavage to make β-subunit core fragment continues throughout pregnancy deactivating hCG [4]. In first trimester pregnancy serum, 9% of hCG dimer molecules are nicked, all free β molecules are also nicked, in the last 2 months of gestation 21% of dimer molecules are nicked and all free β molecules are nicked [39,78]. In pregnancy urine samples, β-subunit core fragment concentration or the degradation end product concentration is relatively small in the first weeks of gestation, it equals hCG dimer concentration at 6–7 weeks of pregnancy. β-core fragment concentrations then exceeds dimer concentrations in urine thereafter, 7 weeks to term [14,89,90]. Mean β-core fragment concentrations average 58% of urine hCG concentrations (mean proportion) at 5 weeks gestation, 105% at 7 weeks gestation rising to 305% of hCG concentrations in the final month of pregnancy [14,89,90]. β-subunit core fragment is the principal hCG related molecule in pregnancy urine samples. In choriocarcinoma cases, 100% hyperglycosylated hCG may be present in serum samples, yet either the hyperglycosylated hCG or β-subunit core fragment may predominate in parallel urine samples [91,92].

All non-trophoblastic malignancies produce primarily hyperglycosylated hCG free β [31,30-34]. In most cases, as a result of macrophage elastase activity at tumor site, the degradation product β-subunit core fragment is detected in corresponding urine samples [14,93-95]. Considering the extremely low levels of hyperglycosylated hCG free β produced by non-trophoblastic malignancies and their rapid clearance from the circulation, urine β-subunit core fragment may be a more sensitive or more measurable tumor marker than serum hyperglycosylated hCG free β [14,93-95].

It is common to find residual nicked hCG or nicked hCG missing βCTP in serum or β-subunit core fragment in urine, the products of degradation, in the weeks following parturition of pregnancy or surgical evacuation of hydatidiform mole or tumor. It is important in these cases to detect nicked hCG and nicked hCG missing βCTP in monitoring serum and β-core fragment in monitoring urine following evacuation of ectopic pregnancy, parturition, evacuation of hydatidiform mole and choriocarcinoma and non-trophoblastic malignancy malignancy to be sure that all tissue is removed.

In the preceding section we examine the structures of regular hCG, hyperglycosylated hCG and hyperglycosylated hCG free β as produced by cells. In this section we show how elastase and other proteases and glycosidase convert 3 forms of hCG β-subunit in serum and urine into 13 forms (Figure 3). Different secreted and degraded forms of hCG may best mark different conditions (Table 1). We emphasize again, it is important when considering a total hCG assay and the structure and degradation findings presented here, to select an assay detecting all form of hCG β-subunit. Regular hCG may mark a normal pregnancy, hyperglycosylated hCG may best mark gestational trophoblastic diseases, hyperglycosylated hCG free β may best mark a Down syndrome pregnancy, nicked hCG missing βCTP may best mark clearing hCG at parturition or following termination, and urine β-subunit core fragment ay best mark non-trophoblastic malignancies.

## Biological functions

### Regular hCG

Placental hCG replaces pituitary LH in controlling progesterone production at the initiation of pregnancy, from implantation of pregnancy (~3 weeks gestation) to 6 weeks of gestation. The syncytiotrophoblast cells make progesterone independent of hCG stimulation from 6 weeks gestation until term. Serum concentrations of hCG, however, rise logarithmically and continuously from implantation of pregnancy to a peak at 10 weeks of gestation (Table 2), levels then fell to about one fifth peak levels and remained at this level to term [14]. hCG concentration seems to have no relationship to their need for promoting progesterone production. That the function of hCG was solely promoting progesterone production, however, was a dogma for almost 80 years (1), even though it was only biologically active for 3 weeks of its 37 week production. This made no biological sense.

**Figure 6:**
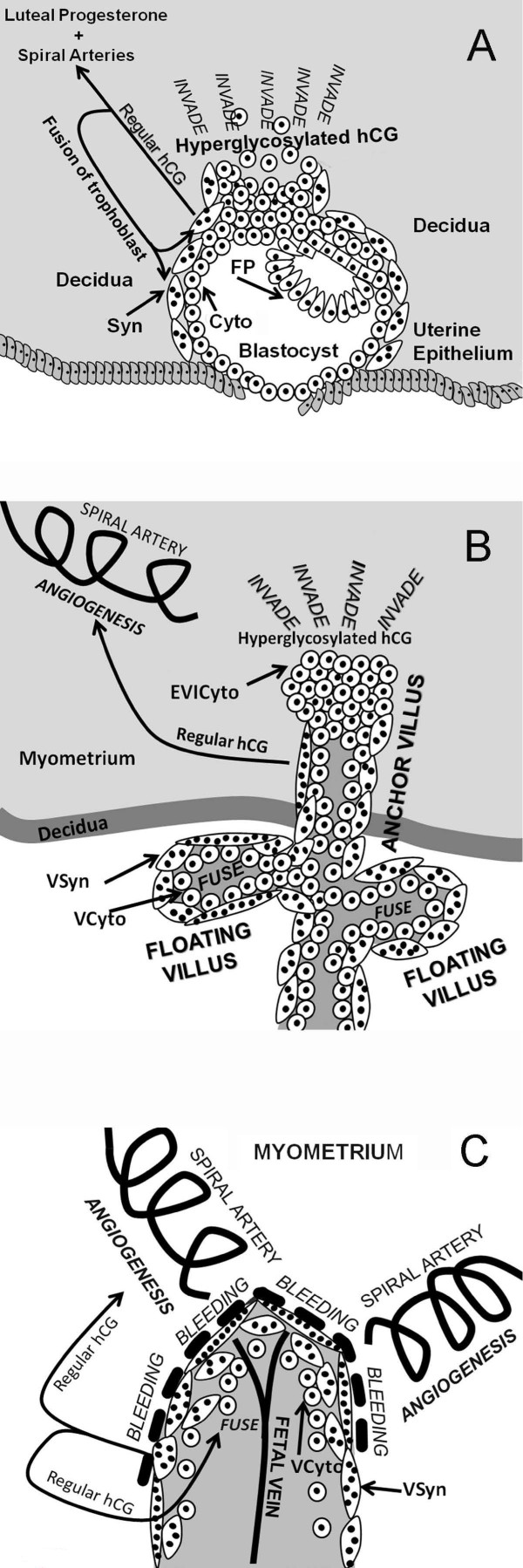
**Villous placental tissue, cytotrophoblast and syncytiotrophoblast cells, and regular hCG and hyperglycosylated hCG function**. Panel A illustrated blastocyst implantation and engulfment and trophoblast invasion at 3–5 weeks of gestation. Arrows illustrates the biological functions of regular hCG and hyperglycosylated hCG. Mononuclear cells are cytotrophoblast cells, cells with multiple nuclei (black circles) represen syncytiotrophoblast cells. In Panel B villous trophoblast formation and function are illustrated at 6–8 weeks of gestation [44,54,114,115]. Figure illustrates villous trophoblast (anchoring villus and floating villus) growth 5 to 10 weeks of gestation, and invasion of the decidua and myometrium in establishing hemochorial placentation. Panel C illustrates functional hemochorial placentation in floating villus at 10–12 weeks of gestation after invasion is complete [114-118]. Cells with varying numbers of multiple nuclei (black circles) represent villous syncytiotrophoblast (VSyn), mononuclear cells are villous cytotrophoblast (VCyto) and extravillous invasive cytotrophoblast (EVICyto). Arrows shows biological actions of regular hCG and hyperglycosylated hCG.

Research by multiple groups in the past 10 years shows a much more logical primary function on hCG, maintaining maternal blood supply to support hemochorial placentation and nutritional support of fetus (Figure 6, Panel A – C) [96-101]. hCG maintains angiogenesis in the myometrial spiral arteries through the length of pregnancy acting on LH/hCG receptors on the spiral arteries [96-101]. It has also been shown that hCG promotes the fusion of villous cytotrophoblast cells to syncytiotrophoblast (Figures 4, Figure 6, Panel A-C) [102]. Both of these biological functions are critical to efficient placentation in humans. This is the more logical prime function of regular hCG through the length of gestation.

### Hyperglycosylated hCG

As discussed, hyperglycosylated hCG is the variant of hCG produced by extravillous invasive cytotrophoblast cells. Examining choriocarcinoma cell lines and testicular germ cell cytotrophoblast cell lines, Cole et al. [41,42] showed that addition of pure hyperglycosylated hCG and not pure regular hCG to first trimester cytotrophoblast cells (villous plus extra-villous cells), and to choriocarcinoma cytotrophoblast cells, promotes growth and invasion across membranes *in vitro*. It also promotes extensive invasion, growth and malignancy by choriocarcinoma cell transplanted into nude mice *in vivo *[41,42] Hyperglycosylated hCG is an autocrine factor produced by extravillous invasive cytotrophoblast cells and acting on these same cells to promote growth of the cells and invasion of other cell by the cells [41,42]. Other studies by Hamade et al. [103] and Lei et al. [104], show that the form of hCG produced by non-villous cytotrophoblast cells (hyperglycosylated hCG) is responsible for promotion of invasion, growth and malignancy in choriocarcinoma cells *in vivo *and *in vitro*, confirming these findings. Multiple articles have confirmed that extravillous invasive cytotrophoblast cells are the invasive cells of the placenta [105-108]. Handschuh et al. [44] confirm that hyperglycosylated hCG is the invasion signal for these cells. Hyperglycosylated hCG has been shown to drive invasion in early pregnancy by extravillous invasive cytotrophoblast cells (Figure 6, Panel A) [42], and to be a critical signal for successful pregnancy implantation (Figure 6, panel A) [43]. As published, deficient hyperglycosylated hCG leads to biochemical pregnancies and pregnancy failures [43,45-47]. All told, hyperglycosylated hCG is produced by extravillous invasive cytotrophoblast cells and acts on these same cells to promote cell growth and invasion, as in implantation of pregnancy and malignancy in cytotrophoblast cell cancers (Figure 6, Panel A).

While it has been shown that hyperglycosylated hCG promotes cell growth through blocking apoptosis [103], only questionable data identifies a receptor for its action. Khoo et al. [109], showed that the TGFβ RII receptor binds a molecule of the size of hyperglycosylated hCG to promote invasion. Structural homology has been noted between the peptide structure of hCG and TGFβ. hCG subunits contain TGFβ cysteine knot structures within the three dimensional core of the molecule [110]. Unraveling of the core as may occur in response to the peptide folding around the larger sugar structures on hyperglycosylated hCG could expose the cysteine knot structures and lead to TGFβ receptor interaction. TGFβ has shown to be critical to extravillous cytotrophoblast cell invasion, implantation and apoptosis, so that the concept of hyperglycosylated hCG as an apoptosis blocker working by antagonizing this receptor seems feasible [111-113]. Research demonstrating the nature of the hyperglycosylated hCG receptor is urgently needed to complete this story.

### Regular hCG – hyperglycosylated hCG partnership

A trophoblast villus is a structural projection of trophoblast tissue, and is the functional unit of trophoblast tissue (Figure 6, Panel B). There are a combinations of anchoring villi and floating villi. Anchoring villi literally anchor villi in the myometrium, they are also the invasive element (Figure 6, Panel B), with extravillous cytotrophoblast cells producing hyperglycosylated hCG driving invasion deeper and deeper into the myometrium [42,43,114,115]. It is floating villi that are active in nutrition transport meeting with the blood from the maternal spiral arteries (Figure 6, panel C) [114,115]. In pregnancy, hundreds of trophoblast villi invade through the deciduas into the uterine myometrium. Figure 6, panel B illustrates 6–8 week gestation villous tissue. It comprises mostly syncytiotrophoblast tissue; beneath these are villous cytotrophoblast cells awaiting fusing into syncytiotrophoblast cells. Regular hCG is made by the syncytiotrophoblast tcells then acts to promote the continuous fusion of villous cytotrophoblast cells to villous syncytiotrophoblast cells (Figure 6, panel B) [55]. Regular hCG circulates in the blood and promotes the growth and expansion of the myometrial spiral arteries to reach the villous tissue and to bleed over the villous tissue. Regular hCG circulates in the blood and promotes the growth and expansion of the myometrial spiral arteries, the source of blood to the villous area after 10 weeks of gestation (Figures 6, Panel B and C).

Throughout the first trimester the villi invade deep into the myometrium, under the influence of hyperglycosylated hCG produced by extravillous invasive cytotrophoblast, reaching one third the thickness of the myometrium. Regular hCG produced by villous syncytiotrophoblast promotes the growth and expansion of the myometrial spiral arteries (Figure 6, Panel B) [115-118]. The villous area starts to be bathed by blood, directly originated from the spiral arteries at around 10 weeks of gestation, the time of the regular hCG and hyperglycosylated hCG peak, competent hemochorial placentation then commences to provide ultra-efficient nutrition to the fetus (Figure 6, panel C) [114-118]. Floating villi are surrounded by syncytiotrophoblast cells form a syncytiotrophoblast membrane. The spiral arteries bleed directly onto the membrane, flooding with blood, nutrients are then very efficiently taken up into the villi and the fetal circulation (Figure 6, Panel C). This is hemochorial placentation [114-118]. Regular hCG continues to be made by syncytiotrophoblast cells on the deeply invaded villous tissue, it promotes continued fusion of trophoblast cells and the continued growth of spiral arteries.

Hemochorial placentation (Figure 6, Panel C) is in many just the end result of a combination of hyperglycosylated hCG driven invasion deep into the myometrium and regular hCG hormonal activity driving the growth and development of the spiral arteries such that they bleed onto the invaded villi. Hemochorial placentation could be considered as simply the consequences of regular hCG and hyperglycosylated hCG action or the product of the regular hCG/hyperglycosylated hCG partnership. As presented in the following section of this review, about evolution, it is the evolution of hCG that seemingly caused hemochorial placentation or efficient placentation to evolve and its advancement in circulating concentration drove it towards the human system, the most efficient of any species [119]. This product of hCG/hyperglycosylated hCG partnership may not be perfect, as present in a later section of this review the high incidence of pregnancy failures in humans is due to deficiencies in regular hCG/hyperglycosylated hCG action [43,45-47,119]. Preeclampsia, a unique human gestational hypertensive disorder is seemingly due to failure of hemochorial placentation and may be caused by deficient hyperglycosylated hCG invasion or regular hCG angiogenesis [119]. Preeclampsia, a unique human gestational hypertensive disorder is seemingly of failure of hemochorial placentation and may be caused by deficient hyperglycosylated hCG invasion or regular hCG angiogenesis [119]. All told this partnership of regular hCG and hyperglycosylated hCG, two oddball variants with the same polypeptide sequences, is critical to human placentation and to the human reproductive process.

### Hyperglycosylated hCG free β

Studies by Acevedo and colleagues show the presence of hCG free β in the membranes of all cancer cell lines and in all histological samples of malignancies [58,120]. Whilst this data is considered rather controversial, new data appears to confirm it in cervical cancer cells although the justifications are somewhat different [121]. Other studies indicate an association between detection of hyperglycosylated hCG free β in serum samples or β-subunit core fragment, its degradation product, in a urine samples in cases with poor grade and advanced stage cancer, or poor outcome malignancy [32,34,122,123]. In a literature review of independent articles investigating free β as a prognostic marker in carcinoma, 12 of the 13 studies demonstrated a clear correlation between expression of hyperglycosylated hCG/hCG free β and poor prognosis [32,59]. These studies collectively indicate that expression of free β invokes a negative outcome in human malignancies either directly, or indirectly, and thus an association between free β expression and malignancy has been proposed. Multiple reports now indicate that free β may have a specific role in malignant transformation of cells [58,61,62,121]. In these, and other studies, direct stimulation of non-gestational malignant cell growth has been demonstrated by the action of free β and where endogenous free β is produced, growth can be inhibited by anti free β antibodies [30,32,38,59,124].

Clearly, free β has a significant role to play in non-gestational neoplasm biochemistry, either as a promoter causing poor malignancy outcome or as an element involved in malignant transformation. Indeed, efforts have been directed toward using different free β derivatives as vaccines in the treatment of non-gestational malignancies. Success has been reported, with free β immunity improving cancer outcome or cancer survival [125-129]. The association of free β detection and poor prognosis, in combination with site specific free β vaccine technology suggests a plausible route to the development of adjuvant cancer therapies specifically targeting patients with free β producing non-gestational tumors.

Both hyperglycosylated hCG and free β promote cancer cell growth, invasion and malignancy [30,32,38,41,42,44,59,124], similarly, both hyperglycosylated hCG and free β function by blocking or antagonizing apoptosis causing cell growth [30,32,59,60,103,121]. In the action of both hyperglycosylated hCG and free β the use of the TGFβ receptor is indicated [30,32,109-114]. As reported, free β is produced by bladder cancer cells and inhibits TGFβ activity in bladder cancer cells [33] and free β-subunit opposes TGFβ growth suppressing functions in bladder cancer cells [32]. It is inferred that both hyperglycosylated hCG and hyperglycosylated hCG free β function similarly, both promoting cell growth, invasion and malignancy by blocking apoptosis through antagonizing a TGFβ receptor.

## Evolution

Chorionic gonadotropin (CG) is part of a family of hormones that includes LH, follicle stimulating hormone and thyroid stimulating hormone. These are the glycoprotein hormones that share a common α-subunit coded by a single gene, and a separate β-subunit which dictates hormone function [130,131]. In 1980 Fiddes and Goodman [130], examined the DNA sequence for the β-subunits of CG and LH and showed that the evolution of CG from LH was by a single deletion mutation in LH β-subunit DNA and read-through into the 3'-untranslated region occurring in early simian primates. In 2002 Maston and Ruvolo [131], examined the DNA sequences of the β-subunit of CG in 14 primates and showed that the genes to make CG and its variants were not present in prosimians or primitive primates (example: Lemur), but evolved by the indicated deletion mutation with early simian primates (example: platyrrhine or new world monkey). Multiple mutation leading to DNA and amino acid sequence changes then occurred with the continued evolution of the CG β-subunit genes from early simian primates to the advanced simian primates (example: pongo or orangutan) and further changes with the evolution of hominids [131]. In summary, prosimian LH has 3 N-linked oligosaccharides mean pI 8.4, from the deletion mutation CG evolved in early simians with 5 oligosaccharides mean pI 6.3, from a further mutation advanced simians evolved a CG with 6 oligosaccharides mean pI 4.8, and with further mutations hominids evolved with 8 oligosaccharides mean pI 3.5 (Table 3).

**Table 3:** Parallelisms between placental implantation and invasion characteristics in primates, presence and sugar structure of CG or LH, and relative brain masses.

Species	Implantation characteristics	Depth of Invasion	Sugar structures, pI	Brain mass (%)
Hominids	Hemochorial	1/3rd myometrium	CG, 8 structures, pI 3.5	2.4%
Advanced simian primate	Hemochorial	1/10^th ^myometrium	CG, 6 structures, pI 4.9	0.74%
Early simian primate	Hemochorial	through decidua	CG, 5 structures, pI 6.3	0.17%
Prosimian primate	epitheliochorial	no-invasion	No CG produced LH, 3 structures, pI 8.4	0.07%

Table summarizes published data [130,131,134,135,137,139,140,146].

The acidity of the evolving CG (and its variants) with additional oligosaccharides very much affects its metabolic clearance rate or circulating half times and thus serum concentration and its effective bio-potency [132,133]. As an example, at one extreme, regular human CG has 8 O-linked and N-linked oligosaccharides all terminating in sialic acid residues. These acidify hCG resulting in a molecule with a mean isoelectric point (pI) of 3.5, and a circulating half time of 35.6 hours [82,84]. At the other extreme, asialo hCG with no acidic sugars on oligosaccharides has a pI of 8.5 and a circulating half time of 5.7 minutes [133]. Because of the acidity due to the sialic acidic residues on each N- or O-linked oligosaccharides, regular hCG circulates for approximately 400 times longer than asialo hCG, raising the circulation concentration proportionately.

The earliest CG form in early simian primates had 2 O-linked oligosaccharides on the β-subunit at serine residues 121 and 132 and one N-linked oligosaccharide at residue 30 [131]. This yielded a molecule with a mean pI of 6.25 [131,135]. As a result of a point mutation at residue 127 (Asn-Ser), the advanced simian primate evolved with a CG having 3 O-linked oligosaccharides [131,134,135], and a mean isoelectric point of 4.9 (Table 1) [135]. With the evolution of hominids, a point mutation occurred at residue 138 and 15 and CG molecules were developed with 4 O-linked and 4 N-linked oligosaccharides [131]. The 4 O-linked 4 N-linked oligosaccharide molecule, is the most acidic CG, pI 3.5 [82]. We deduced from isoelectric points that the 2 and 3 O-linked oligosaccharide 3 N-linked oligosaccharide CGs produced by early and advanced simian primates had minimal and middling circulating half times within the wide half time range of LH at 25 minutes and human CG at 35.6 hours. It is concluded that with evolution from early simians to advanced simians to humans, and the stepwise increases in isoelectric point that circulating half-times and blood concentrations of CG and hyperglycosylated CG increased logarithmically.

Brain size in mammals is directly related to the combination of body mass and the metabolic support of the developing progeny [136]. The greater brain size, seen in advanced primates and hominids, correlates with disproportionately large energy demands by the developing fetuses [136-142]. Numerous studies support the concept that advanced primates, and to a greater extent humans, have had to develop ultra efficient placentation mechanisms to support the increasing nutritional demands of the embryonic brain (Table 3) [136-146].

As shown in Table 3, the prosimian primate had an average size mammalian brain, 0.07% of body mass. In this species, epitheliochorial placentation was sufficient. This involves the placenta loosely attaching to the wall of the endometrial stroma with no invasion. Nutrients and oxygen had to diffuse through multiple myometrial and endometrial stromal layers to transfer from the maternal to fetal circulation. Hemochorial placentation started with the early simian primate with the evolution of CG [137,139,140,146]. In all likelihood regular CG and hyperglycosylated CG initiated and drove hemochorial placentation [119]. The earlier simian primate had 2 O-linked oligosaccharides and 3 N-linked oligosaccharides, was not very acidic, so probably cleared the circulation quickly leaving extremely low concentrations of circulating molecules to promote invasion and angiogenesis, leading to inefficient hemochorial placentation. The placenta invaded through the thickness of the decidua only. Placentation supported nutritional transfer necessary for a brain of 0.17% body mass or 2.5 fold greater than prosimian primates (Table 3) [137,139,140,146]. The advances simian primate had 3 O-linked oligosaccharides, was more acidic, so cleared the circulation at a mediocre rate leaving mediocre concentration of circulating molecule to promote invasion and angiogenesis. This permitted invasion through the decidua to one tenth the thickness of the myometrium [137,139,140,146]. Placentation supported nutritional transfer necessary for a brain of 0.74% body mass or 4.5 fold greater than the early simian brain (Table 3) [137,139,140,146].

Placentation and nutrition transfer was taken to the extreme in the hominids. Hominid CG is very acidicwith 4 O-linked and 4 N-linked oligosaccharides with a circulating half-time of 35.6 hours leading to highly elevated levels of regular CG and hyperglycosylated CG. This led to invasion through the decidua to one third the thickness of the myometrium and to ultra efficient angiogenesis. Placentation supported nutritional transfer necessary for a brain of 2.4% body mass or 3 fold greater than that of advanced simians (Table 3) [137,139,140,146].

Considering the relationship between regular CG, hyperglycosylated CG and hemochorial placentation, and between advancing acidity of CG and advancing invasion and angiogenesis, it would not be unreasonable to propose that the evolution of CG in early simians started primates on the evolution path to advanced brains, or is at the root of the evolution of humans.

The evolution of hyperglycosylated hCG free β appears to an end result result of the evolution regular and hyperglycosylated hCG. The gene for the β-subunit evolved in the human genome. The gene for the α-subunit existed separately as the α-subunit of glycoprotein hormones. When cancer cells regress and retro-differentiate they express cytotrophoblast proteins. By default hyperglycosylated hCG free β is expressed which promotes cancer growth and malignancy [30,32,38,58,61,62,59,124] leading to poor prognosis [32,34,122,123].

## Consequences of evolution

The human CG- and hyperglycosylated CG-driven placentation model may be optimal for fetal nutrition and brain development. In many respects placentation efficiency has expanded to the extreme to accommodate humans supporting a 2.4% body mass brain versus a 0.07% brain in prosimians and many other mammals. Failure of this extreme process, however, may be associated with life threatening complications. Ineffective invasion into the myometrium leads to inefficient hemochorial placentation. This leads to nutritional deficits and anoxia to the fetus compensated for by preeclampsia in the mother [138,142,144,145,147,148]. Preeclampsia has been shown to be linked to unduly low hyperglycosylated hCG levels at the time of initiation of hemochorial placentation at the end of the first trimester [149]. Preeclampsia is one of the most life-endangering complication of pregnancy and occurs uniquely in humans with the fetal demand for deep implantation and the oxygen and nutritional mechanisms needed to support brain development.

With the implantation demands of the fetus, pregnancy failures occur more commonly in humans that in any other species. Pregnancy failures, miscarriages and biochemical pregnancies or early pregnancy losses, account for 41% of gestations in humans compared to rodents (10%) and to a similar low percentage in all other species [43,145,150]. Shallow implantation or ineffective invasion leads to human pregnancy failures [145]. Approximately two thirds of human failures can be attributed to inappropriate invasion [145] and are associated with unduly low hyperglycosylated hCG concentrations [45,73,151]. All pregnancies with normal term outcome produce a significant proportion of hyperglycosylated hCG at the time of implantation [43]. Similarly, two thirds of pregnancies that fail are associated with significant low hyperglycosylated hCG concentrations [43]. Together this data, and the established invasion promoting function of hyperglycosylated hCG suggest that the two thirds of failures due to placentation are the same as the two thirds due to low hyperglycosylated hCG. It is inferred that the high incidence of pregnancy failures, in humans, is a consequence of deficient hyperglycosylated hCG.

Hydatidiform mole does occur in primates [152,153]. Invasive moles, gestational trophoblastic neoplasms or choriocarcinoma, however, have only been observed in humans. These are malignancies of trophoblast tissue associated with extravillous invasive cytotrophoblast cells, driven by the production of CG-H [41,42,146-149]. It appears that just as hyperglycosylated hCG promotes invasion of by normal pregnancy extravillous invasive cytotrophoblast cells during implantation, it is also responsible for the malignancy-like invasion by cytotrophoblast cells in trophoblastic neoplasia. Circulating levels of hyperglycosylated CG are significantly raised by acidic glycosylation, from early simian to advanced simian primates, and then raised further by acidic glycosylation with the evolution of hominids. We deduce that only humans have circulating hyperglycosylated CG levels at magnitudes that can cause malignant-like states. It is inferred that invasive gestational trophoblastic diseases occurs uniquely in humans as a complication of the hominid generation of high concentrations of the invasion promoter hyperglycosylated CG

Considering the relationship between hyperglycosylated hCG and trophoblast invasion, and the proposed link between evolution of hyperglycosylated CG and primate and human evolution, a different perspective emerges on problems with human fecundity due to pregnancy failure, on preeclampsia in human pregnancy and on invasive gestational trophoblastic diseases. Do these findings and hypotheses open new avenues for preventing pregnancy failure and preeclampsia, and for treating or preventing invasive gestational trophoblastic disease?

Is it conceivable that pregnancy failures can be prevented by administration of human CG-H at the time of implantation. The abundance of research showing a link between hyperglycosylated hCG and pregnancy failure supports such trials [43,73,151]. The lack of availability of a pure or clinical usefully preparations of hyperglycosylated hCG at the present time, however, limits the initiation of such trials.

We question similarly the potential use of hyperglycosylated hCG therapy in reducing the risk of preeclampsia and gestation induced hypertension in high risk pregnancies. It is possible that administration of hyperglycosylated hCG at 10–13 weeks of gestation would ensure appropriate completion of invasion and appropriate activation of hemochorial implantation. Risks, such as those which may cause over-invasion or placenta percreta would need to be taken into account. The possible prevention of these disorders could have significant effect on pregnancy complications.

The relationship between human hyperglycosylated hCG and invasive gestational trophoblastic disease is widely proven [41,42,146-149]. Therapy would need to involve the blocking of hyperglycosylated hCG. This may be very much simpler than administrating the invasion-promoting agent. Three separate studies using nude mice transfected with choriocarcinoma cells have shown that blocking hyperglycosylated hCG (choriocarcinoma hCG) production with hyperglycosylated hCG antibody or genetic methods, halts all growth and development of malignancy [41,103,104]. The next step is to perform analogous studies in humans, using human antibodies to hyperglycosylated hCG or vaccines to similarly block malignancy. These would initially be used in patients with chemotherapy resistant cases of choriocarcinoma, and then later examine the prevention of invasion or malignancy in patients with hydatidiform mole.

Hyperglycosylated hCG free β is produced by most human malignancies solely as a consequence of the evolution of regular and hyperglycosylated hCG for promotion of hemochorial placentation. When cancer cells regress and retro-differentiate they express cytotrophoblast proteins. By default hyperglycosylated hCG free β is expressed which promotes cancer growth and malignancy [30,32,33,58,61,62,59,124] leading to poor prognosis [32,31,122,123]. Efforts have been directed toward using different free β derivatives as vaccines in the treatment of non-gestational malignancies. Success has been reported, with free β immunity improving cancer outcome or cancer survival [125-129]. The use of site specific free β vaccine technology suggests a plausible route to the development of adjuvant cancer therapies specifically targeting patients with free β producing non-gestational tumors. An hCG β-subunit derivative vaccine is commercially available (CG Therapeutics, Seattle, USA). It is being tested in clinical trials with different non-gestational neoplasms in the USA.

## Pituitary hCG

In 1976 Chen and colleagues [154], identified human chorionic gonadotropin (hCG) in the circulation of non-pregnant females. This was later demonstrated as being of pituitary origin [155]. Multiple studies with post-mortem human pituitary extracts showed that they all contained hCG at an average concentration of 3.0% of the LH concentration (average 0.8 μg per gland) [156,157]. Individual pituitary glands contained varying concentrations of hCG, 0.05 to 3.3 μg/gland [156,157]. These findings have been confirmed multiple times, with pituitary hCG production being demonstrated as part of normal reproductive physiology [43,157-164]. When considering that hCG and LH share a common α-subunit gene [165], and the LH β-subunit gene is buried amidst 7 hCG β-subunit genes [165], it may be considered as no biological surprise that the pituitary produces a small amount of hCG along with higher concentrations of LH. Pituitary hCG has been detected aside LH in the menstrual cycle [158-162], and post-menopausal in response to the absence of estradiol feedback regulation [43,162-164].

Cole and colleagues recently examined regular hCG in 371 menstrual cycles (Table 4) [166]. The menstrual cycles averaged 28.6 ± 3.8 days (mean ± standard deviation) in length, with an LH peak detected at 14.6 ± 3.1 days following the commencement of menstrual bleeding. The LH peak averaged 210 ± 161 IU/L in magnitude in urine samples (Table 4). Regular hCG was detected (sensitivity 1 mIU/ml) around the time of the LH peak in (LH peak +/- 4 days) in 332 of the 371 menstrual cycles (89%) (Table 4). The average hCG concentration was 1.52 ± 0.91 IU/L in positive LH peak urine samples. The majority of positive regular hCG urines corresponded to the LH peak day, with lesser numbers of urines corresponding to 1 to 4 days before and 1 to 4 after the LH peak. A correlation was observed between LH concentration and the corresponding hCG concentration among the 332 urines, equating hCG and LH by linear regression, r^2 ^= 0.97; by Bartholomew's test of ordered means, p = 0.004 [166].

**Table 4:** Production of LH and regular hCG during non-gestational menstrual cycles [166].

Number of valid non-gestational menstrual cycles	371 cycles
Timing of LH peak concentration during valid menstrual cycles mean ± SD)	14.6 ± 3.1 days
Number of urines tests	11,122
Length of menstrual cycle, start of bleeding to next start of bleeding	28.6 ± 3.8 days
Of valid cycles, the mean peak LH concentration ± SD	210 ± 161 mIU/ml
	
Number of positive daily hCG tests, 1 IU/L sensitivity, in valid menstrual cycles	371
Proportion of menstrual cycles with positive regular hCG test, 1 IU/L sensitivity	332 (89%) cycles
Cycles with positive regular hCG test, regular hCG mean ± SD	1.52 ± 0.91 mIU/ml
Cycles with positive hCG test, hCG range	1.0 – 9.3 mIU/ml

hCG assay used is the Siemens Immulite hCG test with < 0.1% crossreactivity with LH. Standard deviation is abbreviated as SD.

It was concluded that 1.0 mIU/ml or a greater concentration of regular hCG was produced parallel to the LH peak in 89% of menstrual cycles [166]. It was inferred that regular hCG was also produced in the 11% remaining menstrual cycles but at levels < 1 mIU/ml. Regular hCG promotion in the pituitary may be incidental, a byproduct of gonadotropin releasing hormone (GnRH) promotion of LH and follicle stimulating hormone (FSH) expression. Regular hCG expression, however, may be purposeful. LH has a very short circulating half life, 0.42 hrs, while hCG has a long circulating half-time 40 hrs [81,167]. In urine regular hCG is approximately 1% of the concentration of LH. In the pituitary gland, however, regular hCG exists at 3% of the concentration LH [156,157]. In serum, 2-fold higher levels of regular hCG are observed than recorded in urine [14], while 4-fold lower levels of LH are observed [168,169]. As such, regular hCG levels in serum may be much more significant than observed in urine (approximately 7% of LH concentration). The serum concentration represents the concentration acting on ovarian cells. Regular hCG may supplement the promotion of ovulation by extending the LH peak range. This suggest that slow clearing regular hCG may also have a function in boosting the rise in progesterone levels during the beginning of the luteal phase of the cycle. We infer that regular hCG is a functional hormone during the menstrual cycle, either in promoting ovulation or promoting progesterone production.

The pituitary processes molecules slightly differently to syncytiotrophoblast cells, It terminates some N-linked sugar structures in N-acetylgalactosamine-sulfate rather than in galactose-sialic acid (Figure 2). The 4 N-linked oligosaccharides on regular hCG produced by the pituitary terminate in N-acetylgalactosamine-sulfate, while the 4 O-linked oligosaccharides are like those made by syncytiotrophoblast cells terminate in galactose-sialic acid [170]. Because of the difference in N-linked oligosaccharide structure and its acidity, pituitary regular hCG is half as biologically potent as syncytiotrophoblast cell regular hCG, due to a shorter circulating half-life [171].

As recorded in all medical text books, LH and FSH levels are controlled by negative feedback to the hypothalamus by estradiol, controlling GnRH pulses during menstrual periods. As also recorded, estradiol feedback becomes reduced during perimenopause years and disappears during menopause. With this reduction, LH and FSH are normally produced at much higher levels without control at these menopause times. What textbooks do not mention, is that hCG produced at the time of the LH peak also becomes more evident in serum in perimenopause and postmenopause [43,162,164,166,172-176].

The USA hCG Reference Service is a clinical reference service specializing in unexplained hCG production and in gestational trophoblastic diseases. Despite the 20+ publications in major medical journals on the normality of positive serum hCG in perimenopause and postmenopause women and in those having had bilateral oophorectomy, physicians continue to be needlessly concerned, considering gestational trophoblastic disease and cancer as possible options. The USA hCG Reference Service has investigated 319 cases with persistent low hCG results in the absence of history gestational trophoblastic disease or other malignancy (Table 5). These included 112 woman producing hCG that was later proved to be of pituitary origin (Table 5). These were 43 woman postmenopause with amenorrhea, ages 50 to 62, with mean serum regular hCG level of 11 ± 6.2 ranging from 8 to 28 mIU/ml. There were 60 woman perimenopause with oligomenorrhea, ages 39 to 59, with mean serum regular hCG level of 9.8 ± 6.7 [172-176] ranging from 2 to 22 mIU/ml. Finally there were 9 women of normal menstrual cycle age that had received bilateral oophorectomy for cancer-related reasons, putting them into amenorrhea, they had a mean serum regular hCG level of 10 ± 7.2 mIU/ml ranging from 7 to 25 mIU/ml [172-176]. As shown in Table 5, of the 319 cases with persistent low hCG results, the concluding diagnoses has ranged from pituitary hCG, non-gestational malignancy, gestational trophoblastic neoplasm, quiescent gestational trophoblastic disease and false positive hCG. As shown, in the USA hCG Reference Services experience, pituitary hCG is the prime cause of persistent low hCG levels [176].

**Table 5:** The USA hCG Reference Service experience, 1997 – 2008 with 319 cases with persistent low positive hCG in the absence of pregnancy or history of gestational trophoblastic disease or other malignancy.

Source of hCG	Menopause Ages > 50	Perimenopause Ages > 40	Menstrual Ages 18 – 40
Pituitary hCG	43	60	9
Non-trophoblastic neoplasm	0	2	9
Gestational trophoblastic neoplasm	0	2	10
False positive hCG test	1	8	82

Medical centers throughout the world demand a serum total hCG test prior to any surgery, prior to many x-ray procedures and to certain drug protocols. The erroneous assumption is made by medical centers id that a total hCG test can only show pregnancy. If a woman is positive in the hCG test she is referred to an obstetrician. Unfortunately few obstetricians are aware of pituitary hCG. If a women in oligomenorrhea or amenorrhea has a positive pregnancy test prior to surgery it can mean multiple months of delays and false alarms before pituitary hCG is identified and the procedures are rescheduled. The USA hCG Reference Services has consulted on 12 cases waiting multiple years for kidney transplants. When the match was found the procedures were cancelled due to positive hCG tests. In each case multiple months passed before the USA hCG Reference Service was contacted and normal physiology pituitary hCG identified. The patients then rejoined the waiting list once more for a kidney transplant. There should be no requirement for an hCG test if a woman is over 45 years old.

Perimenopause can be difficult to diagnose. The normal symptom is oligomenorrhea. Is the hCG of pituitary origin? A recent article by Granowitz and colleagues [177] shows that the presence of an elevated FSH levels of > 30 mIU/ml justifies the presence of peri- or postmenopause and the presence of pituitary hCG. This is and evaluable method for determining whether pituitary hCG is possible. The pituitary as the source of hCG can be confirmed by placing subjects on a high estrogen oral contraceptive pill for 3 weeks. It will suppress pituitary hCG and the hCG will no longer be detectable [174-176].

## hCG immunoassays

The discovery of monoclonal antibodies in 1975 was paramount to the development of modern immunometric tests [9]. Modern two-antibody immunometric assays for hCG arose in the early nineteen eighties; with them came the concepts of antibody enzyme labeling and high sensitivity fluorimetric and chemiluminescent detection assays [10-14]. These mechanisms are the basis of the automated tests used in all commercial laboratories today. The principal of the immunometric test is one antibody (called the capture antibody) binding one site on hCG and immobilizing it and a second antibody labeled with enzyme tracer (called a tracer antibody) binding a distant site on hCG labeling the immobilize complex. The immobilized and labeled *capture antibody-hcg-tracer antibody *complex can then be quantified, with the amount of tracer or tracer enzyme products being directly proportional to the amount of complex or concentration of hCG. The dual antibody immunometric technologies are also the basis of all modern physician's office point of care (POC) rapid pregnancy tests and home use or over the counter (OTC) rapid pregnancy tests with one antibody immobilized in the result window on the nitrocellulose device and one antibody mixing with the serum or urines and labeled with a blue or red dye as tracer. A positive result is indicated by a line formed by the *immobilized antibody-hCG-dye antibody *complex.

As discussed throughout this article there are multiple variants of hCG in serum and urine in different pregnancy and cancer conditions (Table 1) [14,27-34,41-43,48], these may be invariably detected by different types and different brands of hCG test. By definition there are today 2 clear classes of hCG immunoassay. Intact hCG assays which may or may not detect all forms of dimer (hyperglycosylated hCG, nicked hCG and regular hCG), and total hCG tests which may or may not detect hCG dimer and its free β-subunit (hCG free β, hyperglycosylated hCG free β, nicked free β, free β-subunit missing the βCTP and β-subunit core fragment). There are also three clear types of hCG tests. There are professional laboratory quantitative serum hCG tests (PRL tests), point of care or physicians office qualitative serum and urine hCG tests (POC tests), and home or over the counter qualitative hCG tests (OTC tests).

Most PRL tests used today are total hCG assays. These most commonly involve an antibody to the β-subunit core structure (tertiary structure formed by β1–92) as tracer antibody combined with an antibody to the βCTP (β92–145) as capture antibody. This insures minimal recognition of LH, which lacks a βCTP. There are 2 major limitations with assays using this antibody combination. Antibodies to the natural βCTP commonly are dependent upon the O-linked oligosaccharide structure on the natural βCTP, since this accounts for a major portion of the molecular weight of this segment. Most assays using this antibody configuration poorly detect hyperglycosylated hCG, so critical for use in early pregnancy detection and in choriocarcinoma management (Table 1), because the immunoglobulin used is against the regular hCG βCTP. While this combination usually detects nicked hCG it does not detect hCG or free β missing the C-terminal segment. Some manufacturers have replaced the antibody to the natural βCTP with an antibody to a βCTP synthetic peptide, β131–145, with no oligosaccharide component. An assay with using an antibody to the synthetic βCTP does not distinguish regular hCG and hyperglycosylated hCG. An alternative configuration used by one manufacturer involves 2 antibodies to different regions of the β-subunit core structure. This rare combination permits detection all 13 dimer and β-subunit variants (Figure 3).

We ask why doesn't every manufacturer try and optimize their hCG assay to detect the 13 variants of hCG? Why do some manufacturers sell a test that detects primarily regular hCG and poorly detect variants, yet claim there test to be an optimal hCG test. While others sell tests that detect the13 of 13 dimer and β-subunit variants claim nothing more? As will become apparent from reading this article, detection of 13 of 13 dimer and β-subunit variants is critical for using hCG as a pregnancy test, gestational trophoblastic disease test and cancer test (Table 1)? Unfortunately all variants of hCG are simply considered as general hCG to physicians, they order hCG tests for pregnancy, gestational trophoblastic disease and cancer cases regardless of the specificity of the test used by the laboratory. Laboratories usually order tests based on their throughput, speed and cost. The problem arises with the FDA, who regulate all hCG tests. Their guidelines are entirely based on rather dated 1960s, 1970s and 1980s information, under which hCG is one molecule and detection of only regular hCG is required. There is no requirement to detect hyperglycosylated hCG, even though we now know today that it is the principal or sole variant of hCG present in serum and urine in early pregnancy [40,42,43,45,46,79,178,179], and no requirement to detect free β-subunit or its degradation derivatives (Figure 3), which we now know is the only form produced by cancers. Modern hCG test do state that the test is only approved for pregnancy detection and is not approved for managing gestational trophoblastic diseases or cancer applications (clinicians are not informed of this, it is solely in a note included in the test ingredient box delivered to the laboratory), even though the use of these application continue at every clinical laboratory, regardless of this limitation and regardless of which hCG test their laboratory uses. In fact, no test is approved by the FDA for gestational trophoblastic disease, Down syndrome screening, detecting failing pregnancies or cancer testing. In many ways gestational trophoblastic disease and cancer patients are left in the cold, with no manufacturer interest in producing an optimal test. The end result of the outdated FDA rules is that manufacturers have no interest and no financial benefit in updating or improving the specificity of their assays. In our experience the only tests made that is appropriate for all applications in the Siemen's Immulite series (Immulite 1000, Immulite 2000 and Immulite 2500, same antibody mix but different platforms designed for different throughput).

We examined the major hCG tests sold in the USA, those accounting for > 1% of the USA market (Table 6). They are all automated total hCG immunometric assays. Each assay is sold in semi-automated formats for low throughput centers, and high throughput fully automated formats for intensive use laboratories. All tests are run from a computerized platform. As shown in Table 6, all assays detect regular hCG and all may or may not detect other hCG variants. The Roche Elecsys series poorly detects anything other than regular hCG and hCG free β, and the Siemens Dimension (used to be Dade Dimension), like the Roche assay, poorly detects hyperglycosylated molecules, so critical to pregnancy. Multiple tests, such as the Baxter Stratus, Siemens AC180, Siemens Centaur and Beckman Access and DXI all inappropriately detect hCG β-subunit (not detected on equimolar basis with regular hCG) and hyperglycosylated hCG free β. The majority of tests do not detect hCG or its free β-subunit missing the C-terminal segment [79,179] (Table 6). Only one assay, that using the 2 antibodies to the β-subunit core structure, detects all pertinent standards. As such, only one assay is useful for all pregnancy, gestational trophoblastic disease and cancer application, this is the Siemen's Immulite series (used to be DPC Immulite series). It should be noted that the author of this review, Laurence A. Cole PhD has no financial, consulting or other conflicts of interest with DPC or Siemens. The finding that the Siemen's assay is the most appropriate is strictly the result of blind unbiased testing at multiple laboratories.

**Table 6:** Ability of the most commonly used automated professional laboratory immunometric hCG tests (all tests used by > 1% of USA market) to measure hCG variants [83,84].

Standard	Abbott AxSym	Baxter Stratus	Siemens ACS180	Siemens Centaur	Beckman Access	Ortho Vitros Eci	Tosoh A1A	Roche Elecsys	Siemens Immulite
Regular hCG	+	+	+	+	+	+	+	+	+
Hyperglycosylated hCG	+	+	+	+	+	+	+	?	+
Nicked hCG	+	+	+	+	+	+	+	?	+
Nicked hyperglycosylated hCG	+	+	+	+	+	+	+	?	+
Nicked hCG missing βCTP	-	-	-	-	-	-	X	-	+
Free β	+	?	?	?	?	+	+	+	+
Hyperglycosylated free β	+	?	?	?	?	+	+	?	+
Urine, β-subunit core fragment	-	-	-	-	-	-	-	-	+

Most commercial tests were evaluated at multiple laboratories. Antigens appropriately recognized by assays (detection of standard varies from calibrated concentration by no more than 25%) are indicated by +. Those invariably detected in a specific assay (result more than 25% different from calibrated concentration) are indicated by ?, and those not detected at all are indicted by -.

As shown in Table 7 most assays, with the exception of the Siemen's Immulite series give widely varying results when used for gestational trophoblastic disease and cancer management. Looking, for instance, at a case of serous ovarian cancer in Table 7 (15^th ^cases from top), results varied from 1 mIU/ml (Baxter Stratus) to 115 mIU/ml (Siemen's Immulite series) dependent on the assay being used. In this case the patient was primarily producing hyperglycosylated hCG free β missing the C-terminal segment which was only appropriately detected by the Siemen's Immulite series assays. Examining a partial mole case (20^th ^case from top), results varied from 9 mIU/ml (Baxter Stratus), to 15 mIU/ml (Beckman Access), to 31 mIU/ml (Siemens ACS180), to 48 mIU/ml (Abbott Axsym), and 235 mIU/ml (Siemen's Immulite series) depending on the assay being used. hCG missing the C-terminal segment was also produced in this case. As shown in Table 7, almost every gestational trophoblastic disease or cancer sample is measured with extreme variability. There is no guide given by laboratories to physicians to indicate which test is used or its limitations. Physicans just assume that the test used at the medical center is good.

**Table 7:** Ability of automated laboratory immunometric hCG tests to detect hCG variants in 30 serum samples from gestational trophoblastic disease and cancer patients.

Diagnosis	Abbott AxSym hCGβ mIU/ml (% median)	Baxter Stratus hCG mIU/ml (% median)	Siemens ACS180 hCG mIU/ml (% median)	Beckman Access hCGβ mIU/ml (% median)	Siemens Immulite hCG mIU/ml (% median)
Partial mole	5 (112%)	2 (53%)	3 (72%)	6 (127%)	4 (88%)
Choriocarcinoma	7 (110%)	3 (49%)	5 (71%)	9 (133%)	6 (90%)
Partial mole	10 (107%)	7 (73%)	7 (70%)	10 (108%)	9 (93%)
Complete mole	16 (117%)	12 (89%)	14 (104%)	15 (115%)	13 (96%)
Complete mole	18 (132%)	6 (42%)	14 (104%)	18 (131%)	11 (84%)
Complete mole	16 (119%)	7 (53%)	11 (81%)	16 (119%)	11 (80%)
Partial mole	26 (110%)	15 (62%)	22 (95%)	25 (105%)	19 (81%)
Complete mole	40 (133%)	15 (51%)	30 (100%)	36 (119%)	29 (96%)
Ovarian dysgerminoma	85 (138%)	34 (55%)	63 (102%)	72 (118%)	47 (76%)
Complete mole	91 (116%)	56 (71%)	88 (112%)	90 (114%)	69 (88%)
Testicular germ cell	102 (124%)	60 (73%)	88 (107%)	92 (112%)	78 (95%)
Partial mole	88 (104%)	59 (70%)	81 (96%)	91 (108%)	81 (96%)
Choriocarcinoma	126 (126%)	78 (78%)	102 (102%)	128 (128%)	101 (101%)
Partial mole	153 (143%)	69 (64%)	103 (97%)	121 (114%)	110 (103%)
Serous Ovarian Cancer	70 (64%)	1 (0.9%)	65 (59%)	67 (61%)	115 (105%)
Complete mole	198 (130%)	106 (70%)	159 (104%)	176 (116%)	146 (96%)
Partial mole	245 (124%)	138 (70%)	188 (95%)	214 (109%)	206 (105%)
Ovarian dysgerminoma	221 (99%)	64 (29%)	226 (101%)	242 (108%)	184 (82%)
Partial mole	291 (127%)	173 (75%)	260 (113%)	256 (111%)	224 (97%)
Partial mole	48 (20%)	9 (3.8%)	31 (13%)	15 (6.3%)	235 (99%)
Complete mole	607 (108%)	453 (81%)	603 (108%)	614 (110%)	560 (100%)
Partial mole	668 (115%)	475 (82%)	725 (124%)	782 (134%)	555 (95%)
Choriocarcinoma	603 (93%)	687 (107%)	955 (148%)	842 (131%)	550 (85%)
Complete mole	717 (94%)	759 (99%)	999 (131%)	924 (121%)	769 (101%)
Complete mole	704 (91%)	666 (86%)	943 (122%)	925 (97%)	751 (119%)
Complete mole	1,109 (131%)	655 (77%)	904 (107%)	972 (115%)	827 (98%)
Partial mole	1,027 (112%)	891 (98%)	1,230 (135%)	1,094 (120%)	930 (102%)
Complete mole	9576 (69%)	7995 (58%)	6378 (46%)	7437 (54%)	16320 (118%)
Complete mole	21515 (42%)	16305 (32%)	17070 (33%)	19005 (37%)	52350 (102%)
Choriocarcinoma	13640 (102%)	12145 (91%)	12970 (97%)	13310 (100%)	14720 (111%)
					
Varies by > 25% median	11 of 30 results	21 of 30 results	10 of 30 results	10 of 30 results	0 results

Blindly determined results are presented, as are percents variation of the median result. The proportion of samples using each tests that varies by > 25% of median results is determined.

^a ^hCG missing βCTP, median determined with assays detecting βCTP (Siemens Immulite, Siemens Dimension and hCGβ radioimmoassay)

Similar specificity problems are seen with POC and OTC assays (Table 8). Most POC tests are for some strange reason intact hCG tests detecting dimer only. Due to the configuration of the antibodies in these devices, they poorly detect hyperglycosylated hCG (Table 8) [48]. Similarly, with one exception, manufacturers of OTC tests make devices which poorly detect hyperglycosylated hCG (Table 8). The one exception is the Church and Dwight First Response and Answer devices that equally detect regular hCG, hyperglycosylated hCG and free β-subunit [180]. It is astonishing that other manufacturers of POC and OTC devices market devices that poorly detect hyperglycosylated hCG since measurement of this molecule is critical to early pregnancy detection. How can these be called pregnancy tests? Once more the blame is placed on the FDA and their very out of date guidelines and the absence of a requirement to detect anything other than regular hCG. They basically permit all manufacturers, regardless of what they produce, to label their devices similarly "greater than 99% accurate." This leads to much misinterpretation and confusion.

**Table 8:** Utility of POC and OTC urine hCG testing devises to detect hCG variants. of β-core fragment by urine POC pregnancy devices.

Device	Regular hCG	Hyperglycosylated hCG	hCG Free β	Hyperglycosylated hCG free β	β-subunit core fragment
A. Point of care devices					
Beckman Icon 25	+	?	-	-	-
Beckman Icon 20	+	?	-	-	-
Beckman Icon II	+	?	-	-	-
Inverness Acceava	+	?	?	?	-
Inverness Clearview	+	?	?	?	-
Mainline Confirms	+	?	-	-	-
Quidel Quick Vue	+	?	-	-	-
Quidel Instant Vue	+	?	-	-	-
					
B > Over the counter devices					
Clear Blue Easy	+	?	+	?	-
Clear Blue Easy Digital	+	?	+	?	-
E.P.T.	+	?	+	?	-
E.P.T. Digital	+	?	+	?	-
First Response	+	-	+	+	-
First Response Digital	+	-	+	+	-

Devices were tested with a sample containing 2.5 μg/L (100 IU/L) pure β-core fragment and no other form of hCG. Antigens appropriately recognized by assays (detection of standard varies from calibrated concentration by no more than 25%) are indicated by +. Those invariably detected in a specific assay (result more than 50% different from calibrated concentration) are indicated by ?, and those not detected at all are indicted by -.

We sadly conclude that the field of hCG assays is full of confusion. This is in large part due to inappropriate FDA regulations. It is important for test and platform purchasers to be weary of these limitations and to choose a test with consideration of all possible applications. It seems that only with laboratories exclusively choosing broad specificity tests like the Siemen's Immulite (a small part of the hCG market in the USA) and the Church and Dwight's First Response or Answer (OTC tests, better than all available POC tests), that the market will change and manufacturer's will be economically forced to produce better quality products. Some centers, like the Charing Cross Hospital, Gestational Trophoblastic Disease Center, in London, UK have little trust in any automated hCG test for their gestational trophoblastic disease and cancer applications [181]. They only trust results from their "in house" hCG β-subunit competitive radioimmunoassay. At the USA hCG Reference Service we will only use the Siemens Immulite. We are referred pregnancy and cancer patients with erroneous hCG results. All too often we are referred cases that have received needless therapy due to faulty hCG results, or missed therapy due to missed recurrence of disease. The specificity problem lingers on year after year, it must be addressed, and can probably only be resolved by the FDA.

### hCG standards

The 3^rd ^International Standard (IS, WHO code 75/537) and 4^th ^IS (WHO code 75/589) have been widely used throughout the world for calibrating and testing all PRL, OTC and POC hCG assays for over 20 years [182]. They are both prepared form very large urine extracts prepared by Canfield and Birken at Columbia University, New York, under contract with WHO to produce hCG-related standards. They are calibrated in biological units, international units per liter (IU/L), somewhat arbitrary and meaningless for their principal use, immunoassay. As prepared, 1 μg of pure urine hCG is equivalent to 9.3 IU or a vial of 40 IU contains 4.3 μg of pure urine hCG [182]. Sequence analysis shows that these pure hCG preparations contain 9% nicked hCG, they also contain varying levels of hCG free β and hyperglycosylated hCG free β and significant proportions (15–18%) of hyperglycosylated hCG [18] and β-subunit core fragment [14].

Multiple articles complain about the limitations of these standards, that two tests may give different results using the standards, depending upon whether they detect nicked hCG or hyperglycosylated hCG [14,179,183]. Other authors find problems with the urine origin of the standards and the differences in urine and serum hCG charge variants [184]. A new purer set of standards was needed.

Standards have also produced by WHO for free β-subunit and free α-subunit. These standards are also calibrated in arbitrary international units where 1 IU/L is 1 μg/L. These standards are somewhat incompatible with hCG standards, since 1 IU of free β represent 0.045 nmol of free β, and 1 IU of hCG represents 0.0029 nmol of hCG. As such, 1 IU of free β contains 15.5-fold more β-subunit than 1 IU of hCG.

New standards have now been prepared using molar units so that measurement of hCG and degradations products can be completely compatible [185]. Efforts were directed at purifyng hCG further to rid the preparation of nicked molecules and free subunits [185]. The new hCG standard is the 1^st ^Reference Reagent (WHO code 99/688). Standards were also prepared for the separate β-subunit (WHO code 99/650), separate α-subunit (WHO code 99/720), nicked hCG (WHO code 99/642), nicked β-subunit (WHO code 99/692) and β-subunit core fragment (WHO code 99/708). All of the new standards are strictly calibrated on a molar basis. Physicians throughout the world, however, are very used to IU/L and to all the different elevated, low and high problem values in IU/L. Unfortunately physicians have generally rejected moving to the more logical molar units. With this rejection, laboratories and manufacturers have unfortunately had to refrain from adopting these new standards.

Problems are noted with these new standards. Firstly, while a standard is available for nicked hCG and nicked β-subunit, two standards with little or no clinical application, no standard was made available by WHO for hyperglycosylated hCG a key placental molecules with numerous clinical applications. We are told that this is due to the absence of a reliable source of hyperglycosylated hCG. As shown in Table 2, hyperglycosylated hCG is very prominent in the first 2–3 weeks following implantation of pregnancy, but become a relatively minor component of serum and urine total hCG thereafter. This has made production of a hyperglycosylated hCG standard difficult, especially considering that urine hCG is collected commercially for extractions (like that used by WHO) during the time of the regular hCG peak or at 7 to 12 weeks of pregnancy. Serum and urine contain a hyperglycosylated hCG free β-subunit in cancer cases and a hyperglycosylated free α-subunit during pregnancy. The standards presented by WHO are a separated hCG dimer α-subunit and a separated hCG dimer β-subunit. These may not be optimal for a free α-subunit or hyperglycosylated hCG free β cancer test and may not match optimal specificity tests. New standards are urgently needed for hyperglycosylated hCG, free α-subunit and hyperglycosylated hCG free β assays.

### False positive and false negative hCG assays

In an immunometric assay a complex is formed, *capture antibody-hCG-tracer antibody*. The two antibodies used may be goat, sheep or rabbit polyclonal antibodies or mouse, goat or sheep monoclonal antibodies. Humans extensively exposed to animals or certain animal byproducts can develop human antibodies against animal antibodies (HAAA), whether immunoglobulins A, E, G or M. In addition, humans naturally generate human anti-human immunoglobulin antibodies that can cross-react with and bind animal antibodies. These are called heterophilic antibodies. Humans with recent exposure to mononucleosis can be prone to develop HAAAs and those with IgA deficiency syndrome often have false positive assay problems due to heterophilic antibodies [186]. Each human antibody is bivalent so if an HAAA or heterophilic antibody is present in a person's serum it can bind and link together the immobilized and tracer antibodies making a *capture antibody-heterophilic antibody-tracer antibody *complex or a *capture antibody-HAAA-tracer antibody *complex. Either way it leads to positive test results. These positive results due to sandwiched of antibodies with HAAA or heterophilic antibodies and not hCG variants are false positive hCG test results. A positive test in the absence of pregnancy has led to many women being wrongly diagnosed with cancer, to a lot of needless surgery and chemotherapy, and to a lot of confusion and misunderstanding [187-192].

To avoid false positive tests, manufacturers incorporate animal serum and excess non-specific animal antibodies into all their test ingredients. An excess of non-specific antibodies overwhelming binds and eliminates the interference of heterophilic antibodies and HAAA in human serum samples.

False positive hCG tests were a big problem between 1999 and 2002, when hundreds of women were wrongly assumed to have choriocarcinoma or cancer (textbooks suggested these diagnoses, oncologist taught these diagnoses when hCG was positive in the absence of pregnancy) because of HAAA or heterophilic antibodies and false positive tests [187-191]. Some had history of gestational trophoblastic disease or other malignancy; others had no history and were simply misdiagnosed with cancer on the basis of a positive hCG test in the absence of pregnancy (oncology teachings at this time). It was this false positive issue and the resulting chaos that led to the need for a specialized hCG assay service and to the start of the USA hCG Reference Service in 1997 [187]. The major problem at this time was the Abbott AxSym hCG test, the most commonly used test in the USA during this period. This test had animal serum as a protector for HAAA or heterophilic antibodies in the diluent but not in the antibody components [43]. As such, when serum was tested undiluted (with no diluent) it led to false positive test results. There were multiple multi-million dollar law suits against Abbott Diagnostics Inc. from young women having false positive hCG test and needless hysterectomies and multiple chemotherapy regimens for assumed cancer or assumed gestational trophoblastic disease. There was also a large class action lawsuit from those who underwent cancer chemotherapy. With the multiple lawsuits professional medical societies wrote warning notes to their members about false positive hCG tests. At the same time, Abbott appropriately and quickly fixed their test. We have not heard about an Abbott AxSym hCG false positive patient since this time.

In recent years the USA hCG Reference Service has been referred just 5–6 false positive serum hCG cases each year, mostly from people being tested with a wide variety of different tests (Beckman Access, Bechman DXI, Baxter Stratus, Ortho Vitros Eci, Roche Elecsys, Tosoh AIA, Siemens Centaur, Siemens ACS180, Siemens Immulite and multiple serum POC tests. It appears that every manufacturer's test in extreme circumstances can give a false positive result. With the suits and widespread publicity most physicians became very aware of the hCG false positive problem. It is rare today for the USA hCG References Service to be consulted about a false positive case involving needless hysterectomy or chemotherapy for assumed gestational trophoblastic disease. Occasionally the reference service proves false positive hCG in a case undergoing therapy for gestational trophoblastic disease, a very confusing situation.

There are multiple studies that can be performed in the laboratory that identify false positive hCG. The first is to show the absence of hCG in the urine. Unfortunately, federal drug administration rules limit POC urine tests to 20 mIU/ml sensitivity. Available tests range from 20 to 50 mIU/ml sensitivity. These are the only urine hCG tests offered in America. Urine hCG tends to vary from twice serum concentration down to one tenth serum concentration, dependent upon liquid intake and diuresis. As such, these tests may only be meaningful if serum hCG is over 150 mIU/ml. There is absolutely no reason why urine cannot be tested on the same platforms as are used for testing serum hCG, giving a meaningful urine result of sensitivity as low as 1 mIU/ml [193]. Urine has been shown to work appropriately for measurement of hCG on multiple immunoassay platforms, on the Siemens Immulite [43], Abbott AxSym [194], Siemens Centaur [195] and Roche Elecsys [196]. A second useful way of identifying a false positive serum hCG result is to send the serum to 2 other laboratories using different commercial assays. If the assay results vary greatly, or are negative in one or both alternative tests, then false positive hCG can be presumed.

False negative problems occur with hCG tests. False negative errors can occur in cases with levels of hCG above 500,000 mIU/ml in a "Hook Effect" [14,197]. Since most hCG test are limited in sensitivity to the pregnancy hCG range (at peak median hCG level is 128,300 mIU/ml) the Hook Effect can falsely show very low hCG results or false negative results (i.e. 1 – 100 mIU/ml) when an extremely high hCG concentration is present due to consumption of all (capture and tracer antibody) antibody binding sites on both antibodies, permitting few or no sandwiches [14,197]. In pregnancy, hCG rarely exceed 200,000 mIU/ml, but in complete hydatidiform mole hCG and choriocarcinoma cases hCG can be as high as 3,704,085 mIU/ml. If a patient has an MRI or ultrasound indicating hydatidiform mole or choriocarcinoma and hCG is reported as negative or < 100 mIU/ml then a Hook Effect needs to be assumed and hCG testing repeated using 1/1000 diluted serum. Physicians should inform laboratories of the need for high dilution when testing new hydatidiform mole and choriocarcinoma cases. Laboratories needs to be informed of need for 1/1000 serum dilution to avoid Hook Effect false negative problem.

False negative problems also occur with OTC and POC pregnancy tests. Both type of tests make FDA approved claims like "greater than 99% accuracy" and "use as early as the day of missing menstrual period" yet may only detect pregnancy in a proportion of individuals at the time of missing the menstrual period [178,180,198]. A negative test at the time of missing menses combined with the advertisement "greater than 99% accuracy" may lead to an erroneous false negative conclusion that one is not pregnant. A person may then avoid pregnancy preparations, continue to consume alcohol and fetotoxic medications. Then the person may find out one month later that she truly is pregnant. The pregnancy may a lead low birth weight or preterm pregnancy or possible fetal alcohol syndrome pregnancy. This would all be a consequence of a false negative OTC or POC pregnancy test, due to poor sensitivity or poor specificity.

## Immunoassay applications

Numerous publication in the past 10 years describe new applications for total hCG tests and for new assays measuring hyperglycosylated hCG alone and hyperglycosylated hCG free β alone.

### Regular hCG and pregnancy testing

The prime use of all hCG tests and the only FDA approved use of hCG is pregnancy detection. Hyperglycosylated hCG tests have been 510(k) approved by the FDA as a regular hCG alternative or as a pregnancy test, hCG free β has been similarly 510(k) pregnancy test approved. Total and intact hCG PRL, POC and OTC tests are used for screening for pregnancy in women who are eager to achieve pregnancy and by those eager to exclude pregnancy after having unprotected intercourse. Possible the biggest use of the hCG test today is the nationwide checking of all women, all ages, for any possibility of pregnancy prior to surgery, prior to medical procedures or prior to X-rays. Quantitative urine hCG tests are now used as part of all profession sports to detect administration of hCG in athletes as a promoter of androgens or anabolic steroids.

As shown in Table 2, serum hCG in early pregnancy is primarily hyperglycosylated hCG [43,45,46,73,178]. In the first few week of pregnancy, mean proportions of hyperglycosylated hCG are higher in urine (3^rd^, 4^th^, 5^th ^and 6^th ^week of gestation, mean hyperglycosylated hCG 92%, 68%, 50% and 25% of total hCG) than in serum samples (3^rd^, 4^th ^and 5^th ^week of gestation, hyperglycosylated hCG 89%, 49%, 36% and 21% of total hCG) [43,45,46,73,178]. Most people are first tested or first test themselves for pregnancy during this early pregnancy period. Realistically, if an hCG test is to be considered a real pregnancy test it needs to detect regular hCG and hyperglycosylated hCG equally. This is not true with at least 2 major immunoassay platforms, the Siemens Dimension and the Roche Elecsys (Table 6). It is also not true with any POC test as described in Table 9, and with most OTC tests (Table 8).

**Table 9:** USA hCG Reference Service experience, concentration of hCG, hyperglycosylated hCG and hyperglycosylated hCG free β (HhCG β) in serum samples in gestational trophoblastic diseases and non-gestational malignancies.

Source	n	Total hCG mIU/ml median (range)	hCG-H mIU/ml molar^1 ^% total hCG ± SD?	HhCGβ mIU/ml molar^1 ^% total hCG ± SD
Complete hydatidiform mole (prior to evacuation)	30	192,995 (24160 – 3704084)	4.9 ± 2.1%	7.1 ± 20%^3^
Partial hydatidiform mole (prior to evacuation)	21	48,900 (11600 – 220114)	3.6 ± 1.7%	5.8 ± 22%^3^
Invasive mole, recurrent mole (at commencement of therapy)	72	869 (24 – 30255)	30 ± 35%	7.7 ± 11%
PSTT (at time of diagnosis)^2^	21	30 (1 – 231)	7.1 ± 13%	61 ± 21%
Highly invasive Choriocarcinoma > 50% hyperglycosylated hCG^2^	17	45,350 (3,020 – 596,600)	98 ± 5%	7.8 ± 8.4%
Invasive Choriocarcionoma < 50% hyperglycosylated hCG	44	4,258 (108 – 10,245)	32 ± 11%	6.4 ± 7.5%
Quiescent GTD (at time of diagnosis)^2^	93	22 (1 – 212)	0.10% ± 0.72%	2.7 ± 8.4%
Other gynecologic malignancies (at time of diagnosis)^2^	14	33 (0.5 – 474)	0.55 ± 1.3%	91 ± 11%

SD is standard deviation. Choriocarcinoma cases are those with and without histology (GTN).

^1^Values are measured in molar units and converted to equivalents of hCG, assuming that 1 ng/ml hCG is 11 mIU/ml (using hCG 1^st ^RR standard).

^2^As measured by USA hCG Reference Service, indicated diagnosis confirmed at biopsy by referring center

^3 ^As published by Van Trommel et al. (45).

hCG changes in nature as pregnancy progresses, becoming primarily regular hCG with hyperglycosylated hCG constituting a minor component of hCG (< 2% of total hCG) in the second and third trimesters of pregnancy (Table 2). In urine samples, β-core fragment predominates as the principal variant of hCG from 7 weeks pregnancy until term [14]. No OTC or POC test detects β-core fragment, they rely on detecting regular hCG. Free β-subunit is also most evident in early pregnancy (Table 1), becoming an extremely minor component of total hCG during the bulk of pregnancy (< 1% of total hCG).

We question when can all pregnancy tests be used to definitively detect or exclude pregnancy? PRL serum hCG tests have published sensitivities of 5, 2 or 1 mIU/ml. Urine and serum POC test have published sensitivities of 50, 25 and 20 mIU/ml and urine OTC tests have published sensitivities of 50, 20 and 6.3 mIU/ml [46,48,179]. On the day of missing menses the median hCG concentration in serum is 239 mIU/ml and in a spot urine (any time of day, no adjustment for creatinine) is 49 mIU/ml [42,180]. The range of hCG concentrations varies immensely at this time, serum hCG varies from < 1 to 3,780 mIU/ml and urine hCG from < 1 to 2,132 mIU/ml, this is because of great variation in timing of implantation of pregnancy, which can occur between the 16^th ^and the 30^th ^days of the menstrual cycle [201-203]. If a pregnancy implants early (due to early ovulation, fertilization and implantation) on the 16^th ^day of the menstrual cycle, for instance, with rapid 48 hour doubling of hCG concentrations the serum and urine values can be in thousand of mIU/ml at the time of missing menses. If, in contrast, the embryo implants late (due to late ovulation, fertilization and implantation) the hCG results can be very low at the time of missing menses in serum and urine. Wilcox and colleagues suggested that implantation can occur as late as 11 days after the predicted date of missing menses [202,203]. This seems hard to believe. New studies have found error with this data and show that implantation occurs only as late as the day of missing menses [201]. Since hCG cannot be detected until implantation has occurred [201-203], hCG levels in serum and urine can be < 1 mIU/ml or undetectable on the day of missing menses. Considering all this data, and the concentration of hCG, no assay can 100% detect or exclude pregnancy until 2–3 days after the day of missing menses.

A further complication of pregnancy detection is that 25% of pregnancies are biochemical pregnancies or early pregnancy losses [43,203-205]. These are pregnancies which fail initial stages of implantation leading to rapid failure. They cause, however, a brief incline of serum and urine hCG concentrations which may cause a pregnancy test to misleadingly be positive at the time of missing menses. This transient hCG can in some cases extend to 4 day after the time of missing menses [43,203-205]. This is a further reason for not performing a pregnancy test as early at the time of missing menses. If the pregnancy test is positive it may be a biochemical pregnancy or early pregnancy loss, and if it is negative it may be nothing more than a false negative due to the sensitivity of the test or a true negative and misleading negative due to a late implanting pregnancy. Unfortunately, the literature included with PRL serum tests indicates use as early as 4 weeks since the last menses, and the literature included with POC tests indicates uses as early as the time of missing menses. OTC tests are now competing with each other to determine pregnancy earlier and earlier, with FDA approved claims like "use as early as 4 days prior to the day of missing menses." All these test, whether professional laboratory, OTC or POC are just vague indications at this time, and should not be considered definitive. In our experience, from published data, a firm pregnancy test result, definitely pregnant or definitely not pregnant result cannot be determined until approximately 5 days after missing menses or to approximately 5 weeks of gestation.

### Hyperglycosylated hCG immunoassays for pregnancy testing, pregnancy failure and trisomy pregnancy

The hyperglycosylated hCG assay has been variably available between 2000 and 2008. In 1999 antibody B152 was generated which specifically detects hyperglycosylated hCG and has no measurable binding activity with regular hCG [99]. The assay and applications were licensed to Quest Diagnostics Inc, they own all rights to the antibody. In 2003 the B152 assay was established on the Nichols Diagnostics Inc. Nichols Advantage platform [200]. It was used for Down syndrome screening at Quest Diagnostics. In 2006 Nichols Diagnostics closed down. Since this time Quest Diagnostists has made a microtiter plate assay available for Down syndrome screening through their clinical laboratory service, They have also offered their hyperglycosylated hCG test for gestational trophoblastic disease and pregnancy applications. It is Quest Diagnostics order code 4823. Quest plans to market the test through a major manufacturer as soon as possible. Availability of the hyperglycosylated hCG test for research labs has been variable, numerous investigators used the Nichols Diagnostics assay and now await the commercial release of a new assay. B152 is the only antibody successfully generated to date. It is a low affinity antibody preventing it's used in OTC and POC tests. B152 is an antibody against hyperglycosylated hCG dimer, B152 assays poorly detects the hyperglycosylated hCG free β. Multiple centers have tried to make a hyperglycosylated hCG specific antibody without success. The βCTP and its hyperglycosylated oligosaccharides are seemingly weak or awkward immunogens. Hopefully, new hyperglycosylated hCG-specific antibodies will be generated in the near future along with new commercial assays,

Multiple studies suggest that hyperglycosylated hCG may be used as an improved test for early pregnancy testing in IVF settings [42,45,73,180]. In IVF procedures, regular hCG is administered to promote poly-ovulation. It takes 2–3 weeks after embryo transfer for the administered hCG to leave the system. It is only after the exogenous hCG has departed the system that endogenous hCG can be measured to demonstrate pregnancy. Hyperglycosylated hCG is the principal form of hCG produced in the first weeks of pregnancy (Table 2). If hyperglycosylated hCG is used as a pregnancy test it will only detect endogenous hyperglycosylated hCG independent of administered regular hCG, permitting pregnancy detection as soon as implantation has occurred or at 3–4 days after embryo transfer. A hyperglycosylated hCG pregnancy test also poorly detects or fails to detect biochemical pregnancies or early pregnancy losses. As published, these pregnancies are seemingly a consequence of insufficient production of hyperglycosylated hCG [45]. These very transient pregnancies are a source of 3–5 days false hope among women eager to achieve to achieve pregnancy. They are poorly or not detected by hyperglycosylated hCG, and are best not detected. Hyperglycosylated hCG is a better pregnancy test than regular hCG for assisted reproductive technology applications. As described below, hyperglycosylated hCG may also show advantages in assessing outcome of pregnancy, failing pregnancy or term pregnancy [45].

The hCG doubling test has been widely used as an indication of pregnancy failure, miscarriage or ectopic pregnancy between 4 and 7 weeks of gestation [206-211]. The hCG doubling test involves measuring serum hCG once and them measuring hCG 48 hours later and determining if serum concentration has doubled during this 48 hour period. It is used in individuals with worry of miscarriage due to older age for pregnancy (> 40), previous history of miscarriage or infertility problems. It is also used in the emergency room along with ultrasound to identify ectopic pregnancy. Ultrasound reportedly is 84% sensitive for detecting ectopic pregnancy [212,213]. The hCG doubling test is then used to confirm the presence of ectopic pregnancy. After the hCG doubling test confirmation methotrexate is used to destroy and abort the ectopic pregnancy or a salpingectomy is performed.

The USA hCG Reference Service reference service hears often from women having the hCG doubling test. For instance: "my hCG did not double in 48 hours, does that mean I am definitely have a failing pregnancy?" "I had a salpingectomy for ectopic pregnancy, pathology showed no pregnancy, can you explain why?" The hCG doubling test is grossly inefficient with a very high false positive rate [214,215]. In our experience with the Infertility Clinic at University of New Mexico Medical Center, the test detected 77% of failures at a 36% false positive rate, indicating a predictive value positive of only 25% or 1 in 4 for failures [216]. Considering the incidence of miscarriages and ectopic pregnancy in the USA, 18% of pregnancies (1 in 5.5 pregnancies), the hCG doubling test is only a fraction better than guesswork [216].

The concept of the hCG doubling test arose in the 1980s and was shown useful at this time. At this time hCG tests were hCGβ RIA which equally detected regular hCG, hyperglycosylated hCG, hCG minus βCTP and hCG free β-subunit. It is our understanding that hyperglycosylated hCG and hCG free β-subunit were the principal parts of the test [216]. Today, with modern automated hCG test that poorly detect hyperglycosylated hCG and free β-subunit the hCG doubling test has become less appropriate. Yet there has been no reduction in use of this test by physicians. The USA hCG Reference Service considers this test a very vague indication (1 in 4 detection of 1 in 5.5 pregnancy detections). When people call with concern about results we tell them that it is just a very vague indication.

Multiple studies by different groups suggest that measuring free β-subunit alone and hyperglycosylated hCG alone are much more accurate predictions test of pregnancy failures [43,45,73,151,217]. Hyperglycosylated hCG can be used on the day of implantation to identify a term pregnancy versus failing pregnancy with 100% accuracy [43]. This test can be used at 4 weeks to 7 weeks gestation to detect 73% failures at 2.9% false positive rate, or has a predictive value positive of 82% [151]. Hopefully this new single blood draw test can replace the hCG doubling test is detecting miscarrying and ectopic pregnancies.

Amniocentesis is an absolute test for pregnancy cytogenetics permitting identification of trisomy 21 or Down syndrome (the most common genetic abnormality) and other aneoploidies, monosomies and trisomies of pregnancy. Amniocentesis, however, is performed from 14 to 26 weeks of gestation, leading to late terminations of pregnancy for those opting to abort. Pregnancy termination is a complication of 1 in 200 to 1 in 400 amniocentesis procedures (dependent on center performing procedure and patient complications). Cytogenetics can be determined earlier in gestation, 9–11 weeks gestation, using chorionic villous sampling. The parental risk for termination in chorionic villous sampling is about 2-fold higher, 1 in 100 to 1 in 200 failures. Risk for Down syndrome pregnancies rises greatly as age advances, occurring in 1 in 1000 pregnancy below age 30 to 1 in 105 pregnancies by age 40 [218], yet most individuals attain pregnancy prior to age 30, so that more Down syndrome fetus occur in pregnant woman younger than age 30. In women younger that 30 with a 1 in 1000 Down syndrome risk it is statistically more appropriate to not take the higher risks of amniocentesis or chorionic villous sampling to determine their cytogenetic outcomes. New test were needed or new supplemental tests were needed to select younger women warranting the risks of cytogenetic screening.

In 1984 Chard and colleagues showed that α-fetoprotein was a limited indicator of Down syndrome pregnancy [219]. In 1987 Bogart and colleagues [41] discovered that Down syndrome pregnancies were associated with 2-fold higher pregnancy hCG levels in the second trimester of pregnancy. Then one year later Wald and colleagues showed that unconjugated estriol was also a useful marker [220]. This led to the use of the combination of the three markers, hCG, α-fetoprotein and unconjugated estriol together as a triple test for predicting those at high risk for Down syndrome pregnancy, or those warranting the risk of amniocentesis, in the second trimester of pregnancy. Improvement came in the sensitivity for detecting Down syndrome pregnancy, with the addition of inhibin A as a fourth marker in a quadruple test [221]. Great improvement came using hyperglycosylated hCG in place of regular hCG in the detection of Down syndrome [52]. The hyperglycosylated hCG-based Down syndrome screening is now run routine at Quest Diagnostic Inc.

In the late 1990s serum markers were first used for first trimester Down syndrome screening, with chorionic villous sampling used to determine cytogenetics in those indicated at risk. Use of hCG free β-subunit was found preferable to regular hCG [222]. In 1992 Nicolaides and colleagues found that fetal nuchal translucency as measured with ultrasound was a powerful indicator of Down syndrome pregnancy [223]. The combination of ultrasound nuchal translucency with laboratory measurement of free β-subunit and pregnancy-associated plasma protein-A (PAPP-A) became a standard combination of tests to assess risk for Down Syndrome fetus. In 2003, hyperglycosylated hCG was used to replace free β-subunit and to improve the sensitivity of the test combination [224]. Today, the nuchal translucency combinations with PAPP-A and free β-subunit and PAPP-A and hyperglycosylated hCG are the principal predictors for Down syndrome screening risk used throughout the world. While a positive test with this combination of markers is the optimal prediction, it still only indicates approximately 1 in 20 chance of having a Down syndrome fetus, and the need for chorionic villous sampling or amniocentesis to give a definitive prediction.

### Total hCG and hyperglycosylated hCG immunoassays for gestational trophoblastic diseases

A complete hydatidiform mole is the product of an empty egg (no haploid set) and an androgenic fertilization and is entirely composed of cysts of villous placental tissue (diploid) [225,226]. A partial hydatidiform mole has an androgenic or dispermic pregnancy with an inactive haploid egg leading to a combination of cysts, normal trophoblast elements and fetal elements (triploid) [227]. The two types of molar gestations are easily differentiated cytogenetically [228]. The occurrence of hydatidiform moles in the United States is approximately 1 in 900 pregnancies [229]. Symptoms of a persistent or invasive mole may follow a pathologically identified mole. They may also follow what was recorded as a spontaneous abortion, without cytogenetics or pathology [230,231].

Patients with a hydatidiform mole commonly present with unusually high serum hCG results (Table 9). Generally, an hCG result of > 100,000 is indicative of hydatidiform mole [232-234]. In our experience at the USA hCG Reference Service [49,71] with 30 complete mole cases pre-evacuation, the median hCG in hydatidiform mole cases was 192,995 mIU/ml and the range was 24,161 to 3,704,084 mIU/ml (Table 9). Regular hCG is the predominant measurable hCG that is secreted by the villous syncytiotrophoblast tissue. Hyperglycosylated hCG accounts for 4.9 ± 2.1% of the measured hCG. It is assumed that this is coming from the extravillous invasive cytotrophoblast cells on anchoring villi. In 21 partial moles the median hCG was 48,900 mIU/ml with a range of 11,600 to 220,114 mIU/ml, only 14% of cases exceeded 100,000 mIU/ml. Only 3.6 ± 1.7% of the total hCG of partial moles were hyperglycosylated hCG. Considering complete and partial mole together, 45% of cases were > 100,000 mIU/ml. The median amount of free β-hCG is 7% and 5% respectively for complete and partial moles, and likely represents the breakdown product of the high levels of regular hCG.

The biggest danger with hydatidiform mole is persistence of disease or invasion by extravillous invasive cytotrophoblast cells which can invade the uterus and other organs. This disease is driven by hyperglycosylated hCG. Invasive mole is a malignancy comprising differentiated villous trophoblast cells. Humans uniquely have invasive gestational trophoblastic diseases. Many physicians consider hysterectomy in women first presenting with hydatidiform mole not interested in having further children. This avoids all chances of invasive disease. Approximately 29% of complete hydatidiform mole cases [233-238] and 6.6% of partial hydatidiform mole cases [238] will need treatment for molar invasion. Approximately 3% of complete mole cases and 0.1% of partial mole case will develop choriocarcinoma or highly malignant invasive trophoblast disease [239,240]. Invasive mole is marked by the significant presence of hyperglycosylated hCG (Table 9). When invasive moles are detected, hyperglycosylated hCG comprises 30 ± 35% of total hCG, making them distinguishable from non-invasive moles. Generally [228,229,233-238], an invasive mole is clinically diagnosed by one of the following behaviors of regular hCG measurements: 1) by a plateau in the decline of regular hCG over three weeks following evacuation of a mole or 2) by an hCG decline that has not fallen below 20,000 mIU/ml over a period on four weeks, or 3) by three rising weekly hCG values after prior undetectable levels. Detection of invasive mole dictates the need for single agent chemotherapy to treat invasive disease. Methotrexate or actinomycin D are the single agent chemotherapy drugs of choice.

Choriocarcinoma is a malignancy of transformed non-villous non-differentiating cytotrophoblast cells (no trophoblastic villous differentiation as observed with Invasive Mole). The transformation limits cytotrophoblast differentiation to syncytiotrophoblast cells so that most cases involve predominantly malignant cytotrophoblast cells. The choriocarcinoma cytotrophoblast cells produce hyperglycosylated hCG which drives growth and invasion. As much as 100% of the hCG produced in choriocarcinoma can be hyperglycosylated hCG (Table 9) [18,23,41,49]. Choriocarcinoma commonly is treated by chemotherapy alone. Without surgery or a biopsy, chistology cannot confirm choriocarcinoma. In these cases the name gestational trophoblastic neoplasm (GTN) is adopted. Choriocarcinoma can follows a normal pregnancy (1 in 20,000 live births) [241], and occurs at a rate of 1 in 33 complete hydatidiform moles or 1 in 1000 partial moles (1 in 30,000 pregnancies) [237-239]. Choriocarcinoma is much more common among Asian people, tribal people in The Philippines, Indonesia and Central Africa [229,242]. Choriocarcinoma cases commonly first present with lung and brain metastasis, with hCG levels extending to greater than 5,000,0000 mIU/ml [243].

Highly invasive choriocarcinoma is unique and different from all other hCG producing diseases in that it is marked by 98 ± 5.0% of hyperglycosylated hCG of total hCG (Table 9). Most cases are marked by 100% hyperglycosylated hCG. With the production of high concentrations of hyperglycosylated hCG or invasive signal in choriocarcinoma it can be the most aggressive and invasive of all malignancies, going from no tumor at parturition of pregnancy to tumor in the brain, liver and lungs in just 6 weeks. While extremely fast growing it responds exceeding well to chemotherapy. Five year survival rates for choriocarcinoma treated with chemotherapy range from 81% to 91% [243,244]. Multiagent chemotherapy with EMA-CO (etoposide, methotrexate and actinomycin D oscillating weekly with cyclophosphamide and onvocin) is the recommended chemotherapy for choriocarcinoma [243,244].

Quiescent gestational trophoblastic disease (quiescent GTD) is a syndrome involving inactive or benign gestational trophoblastic disease. It comprises solely or predominantly of highly differentiated syncytiotrophoblast cells. It has minimal extravillous villous or cancer cell cytotrophoblast cells so lacks hyperglycosylated hCG the invasive signal (Table 9). Quiescent GTD was described by the USA hCG Reference Service in 2002 [245-247], its existence and tumor marker parameters were later confirmed independently in the USA and UK [248,249]. Chemotherapy is ineffective during the quiescent phase since the cells are very slow growing, it is recommended to avoid chemotherapy when quiescent GTD is diagnosed [49].

Quiescent GTD commonly self resolves with the disappearance of hCG over 6 months. The USA hCG Reference Service knows of 3 cases, however, that perused with no change in plateau hCG level over 2 years. The USA hCG Reference Service has consulted on 93 cases of quiescent GTD (Table 9). In 20 cases (22% of cases) quiescent disease preceded, however, to active invasive disease. The majority of these cases occurred in patients previously treated for choriocarcinoma or GTN. In these cases, rising hyperglycosylated hCG became evident prior to a significant rise in total hCG, which was the first evidence of active disease [49]. Considering the 22% risk of progression to active disease, it is critical to monitor hCG regularly in quiescent GTD cases. We recommend that woman with quiescent GTD be placed on an oral contraceptive pill and avoid achieving pregnancy, at least until hCG has been undetectable for 6 months.

Many of the cases referred to the USA hCG Reference Service were inadvertently given chemotherapy for assumed invasive mole or persistent disease. Half of the 101 cases received needless chemotherapy, multiple regimens of chemotherapy or hysterectomy for assumed active disease. We know of no case in which the therapy appropriately suppressed disease (hCG < 1 mIU/L). The failure of chemotherapy to eradicate hCG producing trophoblasts is likely because of the lack of proliferation of the inactive quiescent disease. We know of one patient who died due to complications of the chemotherapy regimens. We conclude that chemotherapy and surgery should be avoided in cases of quiescent GTD. We also infer that it is important to consider quiescent GTD when a mole, GTN or choriocarcinoma case demonstrates an hCG plateau below 215 mIU/ml before commencing chemotherapy. If a hyperglycosylated hCG test is not available, then the persistence of total hCG results, permitting a 2 fold natural variation, should be monitored and considered as evidence for inactive or static disease. It should noted that < 212 mIU/ml represents a miniscule trophoblast cell mass, and that > 2,000 mIU/ml of hCG in serum is required before a tumor could be seen by MRI [240].

### Hyperglycosylated hCG free β immunoassays and placental site trophoblastic tumor (PSTT)

PSTT is a malignancy of of so called intermediate trophoblast cells, syncytiotrophoblast cells with 3–5 nuclei). As such, it does not produce hyperglycosylated hCG. It is a very rare condition accounting for 1 in approximately 40,000 pregnancies. It also has a poorer prognosis than choriocarcinoma [250,251]. The five year survival rate for PSTT is 74% [250], compared with 91% for choriocarcinoma [244]. Classically it presents as a tumor in the uterus, which is readily removed by hysterectomy.

The range of regular hCG in PSTT cases, if present, varies substantially, reportedly between 6 to 107,000 mIU/ml. The USA hCG Reference Service has consulted on 7 PSTT histology-proven cases. It was noted that all primarily produced hyperglycosylated hCG free β-subunit rather than regular hCG or hyperglycosylated hCG, even though these tumors involve syncytiotrophoblast cells. As discussed in this review article, hyperglycosylated hCG free β acts like hyperglycosylated hCG as a promoter of cancer cell growth and invasion. It was suggested by the USA hCG Reference Service that the proportion of hyperglycosylated hCG free β (> 30% of total hCG) may be an absolute marker for discriminating choriocarcinoma and PSTT [252]. Since this time the USA hCG Reference Service has used an hCG free β tests to show that 14 further cases were PSTT and not choriocarcinoma [253,254]. This was later confirmed by expert pathology un the month that follow. Reviewing all 21 cases referred to the USA hCG Reference Service to date, the median regular hCG was 30 mIU/ml (range 1 – 231 mIU/ml). The proportion of hCG free β averaged 61% of the total hCG concentration (Table 9).

PSTT requires a very different treatment protocol to GTN and choriocarcinoma. As such, it is important to make sure that the diagnosis is correct. The hCG free β subunit tests is widely available at clinical laboratories for Down syndrome screening (results presented as multiple of median). Special arrangements will need to be made with laboratory to obtain a concentration for cancer assessment. Results are normally determined in nanograms per milliliter. One nanogram per milliliter is equivalent to 18 mIU/ml.

### Hyperglycosylated hCG free β immunoassays and cancer

It has long been known that most non-trophoblastic malignancies in advanced stages produce the hyperglycosylated hCG free β. Early reports note free β production by cervical cancer, breast, bladder, ovarian, brain, colorectal, uterine, brain and lung malignancy cell lines [30,122,255-261]. As reported by Acevedo et al. [58] and by Regelson [28], almost every human malignancy generates hyperglycosylated hCG free β. This claims is somewhat debatable, and at best the diagnostic potential for even the most recognised free β producing tumors is around 38% (Table 10). However, more recent studies clearly show that while the hyperglycosylated hCG free β is not a reliable diagnostic marker or a good marker in these non-trophoblastic malignancies it does have value as an excellent marker for poor prognosis (24). Hyperglycosylated hCG free is terminally degraded to generate β-subunit core fragment, β6–40 disulfide linked to β55–92, with degraded oligosaccharides(Figure 3), a more sensitive tumor marker in urine samples (Table 11) [32,262]. Serum hyperglycosylated hCG free β and urine β-subunit core fragment have both proven useful in the detection and management of a wide range of malignancies [32,256,262,263,265-270]. As shown in Tables 10 and 11, 47% of 558 malignancy cases tested for urine β-subunit core fragment and 30% of 1202 malignancy cases tested for serum free β were positive. The expression of the free β or detection of β-subunit core fragment in a patient with malignancy correlates with poor grade and stage of tumor [31,59], indicating that expression of free β is associated with a negative outcome in human malignancies.

**Table 10:** Use of serum free β-subunit as a tumor marker for detection of malignancies.

Malignancy	Patients	Sensitivity (> 3 fmol/ml)
Ovarian cancer	150	38% average of 3 reports
Cervical cancer	60	37% average of 2 reports
Endometrial cancer	55	33% average of 2 reports
Vulvar	50	38% single report
Bladder cancer	99	38% average of 2 reports
Lung cancer	177	18% average of 2 reports
Colorectal cancer	436	17% average of 2 reports
TOTAL	1152	30% average of 16 reports

Averages are determined from multiple reports by combining total cases positive for free β-subunit by total cases tested.

**Table 11:** Use of urine β-subunit core fragment as a tumor marker for detection of malignancies.

Malignancy	Patients	Sensitivity (> 3 fmol/ml)
Healthy no history of malignancy	97	9.3%
Healthy benign disease	159	6.9%
TOTAL	256	7.8%
		
Ovarian cancer	106	68%
Cervical cancer	89	46%
Endometrial cancer	110	51%
Pancreatic cancer	29	55%
Bladder cancer	102	48%
Lung cancer	122	24%
TOTAL	558	47%

Examining Tables 10 and 11, with few exceptions, gynecologic and urinary tract malignancies appear to be the biggest producers of free β. Free β is detected in serum in 38% of ovarian malignancies and β-subunit core fragment in the urine of 68% of malignancies. Similarly, free β is detected in the serum of 38% of bladder malignancies and β-subunit core fragment in the urine of 48% of malignancies. In contrast, free β is detected in the serum of just 18% of lung cancer cases and β-subunit core fragment in the urine of just 24% of malignancies. Serum free β and urine β-core fragment are very non specific markers, detecting a proportion of all malignancies. The urine β-core fragment test may be useful as a general screening test for malignancies used at annual physical examination or in a life insurance examination detecting 47% of malignancies. A positive test, however, would need to be followed by a complete body malignancy scan (MRI scan of head and pelvis and CT scan of chest) to detect the malignancy.

We question why non-gestational malignancies produce hyperglycosylated hCG free β while choriocarcinoma makes the hyperglycosylated hCG dimer? Research indicates that in general, hCG dimer is associated with high β-subunit production, 1 mIU/ml to > 5,000,000 mIU/ml dimer in blood, observed in trophoblastic neoplasms and choriocarcinoma, while hyperglycosylated hCG free β is associated with low β-subunit production, 1 – 200 mIU/ml in blood, by non-trophoblastic malignancies [49,31,252]. This is consistent with concentration and the law of mass action. In contrast, during very early pregnancy, and in quiescent and recurrent choriocarcinoma and gestational trophoblastic neoplasms, low concentrations (0.1 – 20 ng/ml) of dimer alone may be produced [43,49]. This argues against the law of mass action being the cause of free subunit productions, and suggests that trophoblast cells may simply be much better equipped for producing α – and β-subunits held as dimer when compared with non-trophoblastic neoplasm cells.

## Conclusion

The field of hCG has changed dramatically in the past 10 years. This is the focus of this review. It has moved from hCG a hormone produced by trophoblast cells that promotes progesterone production by luteal cells to hCG as group of molecules each with different functions. That is regular hCG a hormone produced by syncytiotrophoblast cells which promotes myometrial spiral artery angiogenesis, aiding nutrient supply to the placenta. Hyperglycosylated hCG is an autocrine factor made by extrauterine invasive cytotrophoblast cells in pregnancy and malignant cell non-villous cytotrophoblast cell is choriocarcinoma and testicular germ cell malignancies. This form of hCG controls placental invasion and implantation and cancer cell malignancy. Hyperglycosylated hCG free β is an invasion promoter made by non-trophoblastic cancer cells which enhances cancer cell growth and malignancy. All of these new findings have been confirmed by multiple separate laboratories. In many ways, the new hCG as we know it today, is a very unique molecule, one α and one β polypeptide structure and 3 independent bioactive molecules. This is a unique biological combination, 3 molecules differing in carbohydrate structure.

Major changes have also occurred in the past 10 years in the hCG immunoassay field, New perspectives on hCG assays and what they should detect. Multiple new application for hyperglycosylated hCG in Down syndrome screening, detecting pregnancy, detecting pregnancy failures and in the diagnosis and management of gestational trophoblastic diseases. New applications for free β in predicting Down syndrome pregnancies, in diagnoses of PSTT and as a tumor marker for all non-gestational malignancies, and new vaccine treatments blocking free β enhancing survival of malignancies.

In many respects the new discoveries are like the rediscovery of hCG as a group of molecules has occurred, a group of molecules critical to human evolution and essential to human pregnancy. With all the new doors opening in the hCG story, everything is still far from complete. Yes, we know that hyperglycosylated hCG is an autocrine factor that controls extravillous invasive cytotrophoblast growth and invasion but we are far from sure what receptor it binds. We know that its mechanism involves blocking apoptosis, but how does it promote metalloproteinases to advance invasion? Multiple indications and preliminary studies suggest a TGFβ-like receptor but this is far from proven. This needs to be resolved. Similarly, we know that hyperglycosylated hCG free β also seemingly binds a cytokine receptor and works through a mechanism involving antagonism of apoptosis, but what receptor does this bind? This also needs to be resolved. The idea evolves of using hyperglycosylated hCG to prevent failure of pregnancy and to prevent preeclampsia. It sounds incredible to consider preventing these complications of pregnancy, but at least ten years of testing in advanced primate and human clinical trials is needed before this can become realistic. Clinical trials are already ongoing using in hCG β-subunit vaccine in the treatment of malignancies. A lot of promise is in store. At the current time we do not have a standard for hyperglycosylated hCG or commercial or recombinant method for producing hyperglycosylated hCG, maybe this is a first step. A lot of improvement is needed in the coming years in hCG assay development, in improving total hCG assays to measure regular hCG, hyperglycosylated hCG and hyperglycosylated hCG free β on an equi-molar basis, and in the development of new hyperglycosylated hCG only assays, using them in IVF pregnancy testing and applying them to pregnancy failures and other promising applications. Yes, the past 10 years has shown great advances in the field of hCG, what the next 10 years may show could be even more exciting.

## Competing interests

The author of this review is a consultant for Quest Diagnostics Inc. a nationwide clinical laboratory selling pregnancy tests and Down syndrome screening tests. He is also a consultant for Church and Dwight Inc. on the design and application of a hyperglycosylated hCG home pregnancy tests. Neither of these consultancies has in any way affected or changed any part of this publication. Dr. Cole has no other commercial interests or shares in any pertinent companies.
